# Systems modelling of the EGFR-PYK2-c-Met interaction network predicts and prioritizes synergistic drug combinations for triple-negative breast cancer

**DOI:** 10.1371/journal.pcbi.1006192

**Published:** 2018-06-19

**Authors:** Sung-Young Shin, Anna-Katharina Müller, Nandini Verma, Sima Lev, Lan K. Nguyen

**Affiliations:** 1 Department of Biochemistry and Molecular Biology, Biomedicine Discovery Institute, Monash University, Clayton, Victoria, Australia; 2 Molecular Cell Biology Department, Weizmann Institute of Science, Rehovot, Israel; Icahn School of Medicine at Mount Sinai, UNITED STATES

## Abstract

Prediction of drug combinations that effectively target cancer cells is a critical challenge for cancer therapy, in particular for triple-negative breast cancer (TNBC), a highly aggressive breast cancer subtype with no effective targeted treatment. As signalling pathway networks critically control cancer cell behaviour, analysis of signalling network activity and crosstalk can help predict potent drug combinations and rational stratification of patients, thus bringing therapeutic and prognostic values. We have previously showed that the non-receptor tyrosine kinase PYK2 is a downstream effector of EGFR and c-Met and demonstrated their crosstalk signalling in basal-like TNBC. Here we applied a systems modelling approach and developed a mechanistic model of the integrated EGFR-PYK2-c-Met signalling network to identify and prioritize potent drug combinations for TNBC. Model predictions validated by experimental data revealed that among six potential combinations of drug pairs targeting the central nodes of the network, including EGFR, c-Met, PYK2 and STAT3, co-targeting of EGFR and PYK2 and to a lesser extent of EGFR and c-Met yielded strongest synergistic effect. Importantly, the synergy in co-targeting EGFR and PYK2 was linked to switch-like cell proliferation-associated responses. Moreover, simulations of patient-specific models using public gene expression data of TNBC patients led to predictive stratification of patients into subgroups displaying distinct susceptibility to specific drug combinations. These results suggest that mechanistic systems modelling is a powerful approach for the rational design, prediction and prioritization of potent combination therapies for individual patients, thus providing a concrete step towards personalized treatment for TNBC and other tumour types.

## Author summary

We applied a systems modelling approach combining mechanistic modelling and biological experimentation to identify effective drug combinations for triple-negative breast cancer (TNBC), an aggressive subtype of breast cancer with no approved targeted treatment. The model predicted and prioritized the synergistic combinations as confirmed by experimental data, demonstrating the power of this approach. Moreover, analysis of clinical data of TNBC patients and patient-specific modelling simulation enabled us to stratify the patients into subgroups with distinct susceptibility to specific drug combinations, and thus defined a subset of patient that could benefit from the combined treatments.

## Introduction

Resistance to anti-cancer drugs is a major clinical problem and a prevalent cause of cancer-related death. Consequently, combination therapy has recently emerged as a powerful strategy to circumvent drug resistance and thus is being actively pursued in many cancers [1]. Yet, we face daunting challenges of how to predict the best combinations of targets and importantly, how to prioritize those combinations for clinical testing. Given the number of possible target combinations are vast but clinical trials are slow and expensive, there is an urgent need to develop rational and unbiased approaches to predict effective drug combinations, prioritize them and stratify patients for optimal benefit.

Analysis of the activity and dynamics of signalling networks should aid in the development of effective combination therapies against oncogenic kinases [2]. However, signalling networks often contain complex pathway crosstalk and feedback regulation, making it difficult to analyse the drug-induced network behaviours and emergent network features using experimental approaches alone [3]. Instead, experimentally-grounded mathematical models of signalling networks that integrate pathway crosstalk and feedback loops provide a powerful quantitative framework for the systematic analysis of network-level dynamics [4–6]. As feedback loops often lead to unexpected adverse effects of drug treatments, these models have the power to predict effective and non-trivial combination treatments in cancer cells [3]. Here, we employ a systems-based approach combining mechanistic modelling and biological experiments to predict and prioritize drug combinations for triple negative breast cancer (TNBC), an aggressive subtype of breast cancer.

While targeted therapies have vastly improved the survival of ER/PR-(Estrogen/Progesterone receptor) and HER2/ErbB2-positive breast cancers, TNBC, which is defined by the absence of these hormone receptors and of HER2 amplification [7], remains a major clinical challenge with no efficient targeted therapy [8]. TNBC comprises an heterogeneous group of cancers with at least six molecular subtypes (BL1: basal-like 1, BL2: basal-like 2, IM: immunomodulatory, M: mesenchymal, MSL: mesenchymal stem-like, and LAR: luminal androgen receptor), which display different clinico-pathological features and divergent responses to treatments [9]. This heterogeneity requires efficient patient stratification strategies, capable of predictively selecting most suitable patients for specific treatments, while excluding the unsuitable individuals or subgroups.

Several signalling pathways, including the PI3K/mTOR pathway, JAK/STAT, Ras/Raf as well as the EGFR and c-Met pathways have been considered as potential therapeutic targets for TNBC [10]. The Tyrosine Receptor Kinases EGFR and c-Met are highly expressed in TNBC and implicated in TNBC progression and metastasis [11–13]. However, their targeting by monotherapeutic agents has a marginal clinical efficacy possibly due to bidirectional compensatory responses, activation of alternative pathway(s), and/or other resistance mechanisms [14,15]. EGFR and c-Met share overlapping downstream signalling pathways and can trans-phosphorylate one another. We have recently found that PYK2 is a common downstream effector of EGFR and c-Met; and have delineated their crosstalk signalling in TNBC, demonstrating that knockdown of PYK2 facilitates receptor degradation and concomitantly inhibits EGF-induced ERK1/2 and STAT3 phosphorylation. PYK2 positively regulates STAT3-phosphorylation in response to EGFR activation, while pSTAT3 binds to the PYK2 promoter and enhances PYK2 transcription, and indirectly upregulates c-Met expression [16]. These results suggest a number of positive-feedback loops between PYK2, STAT3, c-Met and EGFR, and imply that co-targeting of components of this circuit could have therapeutic impact.

Here, we develop and experimentally validate a quantitative mechanistic model of the EGFR-PYK2-c-Met interaction network and demonstrate the utility of this model in accurately predicting and importantly, prioritizing synergistic drug combinations for TNBC. Notably, model predictions supported by *in vitro* and *in vivo* data reveal co-inhibition of EGFR-PYK2 and EGFR-c-MET yield the most synergistic effects among other possible drug combinations. Moreover, integrated modelling-experimental analysis uncovers switch-like response to single-drug inhibition at both the signalling (STAT3 and ERK activation) and cell proliferation levels, and establish a link between these switches and the drug synergy displayed by the combination treatment. Excitingly, simulations of patient-specific models generated through incorporating gene expression data from individual TNBC patients led to stratification of patients into subgroups with distinct susceptibility to EGFR-PYK2 co-inhibition, thereby predicting a subset of highly susceptible patient characterized by high PYK2 expression. Collectively, we present a proof-of-concept study demonstrating the power of quantitative mechanistic modelling in the rational discovery and prioritization of effective drug combinations for TNBC. Importantly, the principles contained in this work are highly applicable to other signalling systems and/or tumour types, which should significantly aid in the selection of best target combinations for further in-depth preclinical and clinical testing.

## Results

### Description of the EGFR-PYK2-c-Met network interactions and mathematical model assumptions

**Fig 1:**
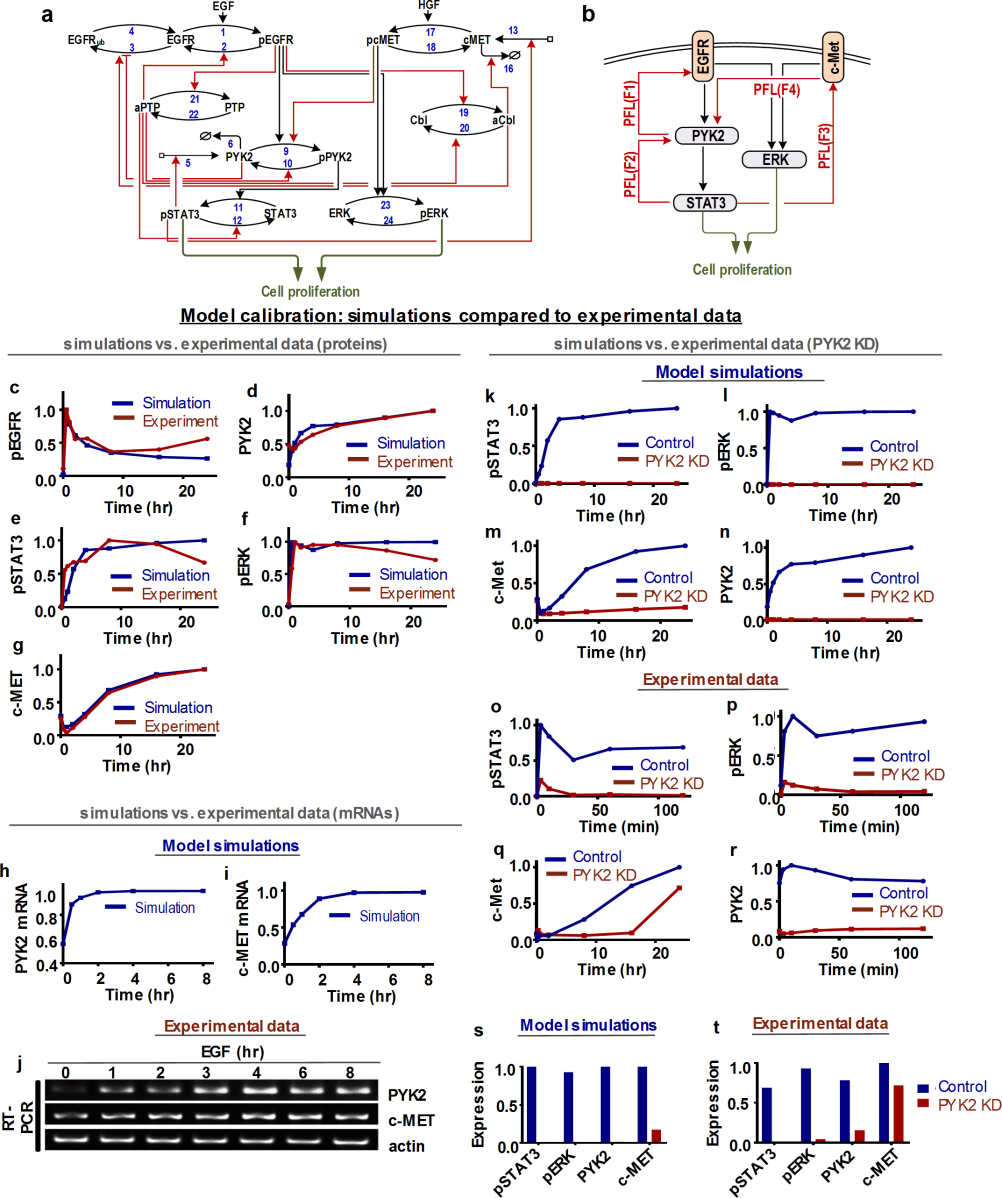
Schematic interactions and data-based calibration of the EGFR-PYK2- c-MET network model. **(a)** Detailed reaction scheme of the kinetic EGFR-PYK2- c-MET model. pEGFR: phosphorylated, active EGFR; EGFR_ub_: ubiquitinated EGFR; pPYK2: phosphorylated, active PYK2; pSTAT3: phosphorylated, active STAT3; pcMET: phosphorylated, active c-MET; aCbl: activated Cbl; pERK: phosphorylated, active ERK; aPTP: activated phospho-tyrosine phosphatase. *v*_*i*_*s* denote the reaction rate numbers whose detailed formula are given in the Supplemental Information (SI). **(b)** An abstracted schematics of the network highlighting multiple interconnected positive feedback loops (PFL). **(c-t)** Model fitting to experimental data. Comparison of simulated (red lines, showing the best-fitting model) and experimentally observed (blue lines) time-courses. **(c-g)** Effect of EGF stimulation for 0.5h, 1h, 2h, 4h, 8h, 16h and 24h on phosphorylated and total protein levels of indicated proteins. (**h-j)** Effect of EGF stimulation for 0.5h, 1h, 2h, 4h, 8h on mRNA levels of c-MET and PYK2. **(k-r)** Effect of PYK2 knockdown on phosphorylated levels of STAT3, ERK and total level of PYK2 upon EGF stimulation for 0 min, 3min, 10min, 30min, 60min and 120min (c-MET for 0h, 0.25h, 0.5h, 2h, 8h, 16h and 24h). All experiments were performed using MDA-MB-468 cells. Simulation (s) and experimental steady state data (t) captured from k-r.

The interconnection between EGFR, PYK2 and c-Met signalling in TNBC cells and the involvement of STAT3 activation in a positive feedback loop [16] led us to examine the dynamic behaviours of the EGFR-PYK2-c-Met signalling network in a quantitative and predictive manner. To this end, we constructed, calibrated and validated a computational model of this network. Reconstruction of the network structure revealed a multitude of interconnected positive and negative feedback loops, suggesting the network is likely to display highly non-linear and emergent dynamical properties. This model is based on ordinary differential equations (ODEs) which incorporates these key feedback interactions as well as other established regulatory mechanisms. A detailed reaction scheme of the EGFR-PYK2-c-Met network is presented in Fig 1A, while a simplified schematic diagram depicting the core signal flow and salient feedback loops is given in Fig 1B. Detailed description of the model reactions, ODEs and parameter values are available in the Supplementary Information (S1–S3 Tables). Below, we describe the mechanistic biological observations and regulatory mechanisms that underlie the key model assumptions built into the model.

### Positive feedback between EGFR and PYK2

We reported recently that PYK2 is a downstream effector of both EGFR and c-Met in TNBC cell lines, and that stimulation of EGFR or c-Met by their cognate ligands EGF or HGF, induced rapid phosphorylation of PYK2 on tyrosine 402 (pY402) and ensuing activation [16] (reaction 9, Fig 1A). On the other hand, PYK2 depletion substantially reduced EGFR level shortly (30–120 mins) after EGF stimulation. These observations suggest that PYK2 either attenuates receptor ubiquitination and consequently its lysosomal degradation (reaction 3) or facilitates receptor deubiquitination (reaction 4). Although we assumed the former to be the case, the model’s dynamic features are not significantly affected even if we assumed the later instead. The reciprocal interactions between PYK2 and EGFR form a positive feedback between these proteins, denoted as F1 in Fig 1B.

### Negative feedback loops involving EGFR

As indicated in Fig 1A, EGFR is assumed to exist in three inter-convertible molecular states: unmodified, phosphorylated (activated) and ubiquitinated (EGFR_ub_); transition between these states are regulated by the relevant enzyme regulators. Upon EGF binding, EGFR undergoes rapid phosphorylation, internalization and ubiquitination by the E3 ubiquitin ligase Cbl, which targets it for lysosomal degradation [17]. As activated EGFR induces phosphorylation (on tyrosine 371) and activation of Cbl (reaction 19) [18], which eventually leads to EGFR degradation, EGFR and Cbl are connected via a negative feedback. For simplicity, we avoid modelling of EGFR synthesis and degradation processes, and instead assume that EGFR molecules targeted for degradation are locked in a dynamic pool of ubiquitinated EGFR (Fig 1A). Similar to EGFR ubiquitination, EGFR phosphorylation is also regulated by a negative feedback loop between EGFR and tyrosine phosphatases, such as SHP-1 (denoted generically as PTP), which are activated by EGFR and induce receptor dephosphorylation and consequently its inactivation (reactions 21 and 2).

### Transcriptional positive feedback between PYK2 and STAT3

By using chromatin immunoprecipitation (ChIP) and luciferase-reporter assay we previously showed that STAT3 directly binds to PYK2 promoter and enhances its transcription in response to EGF stimulation in TNBC cells. We further showed that STAT3 inhibition by Stattic or AG490 (JAK/STAT3 inhibitor) significantly reduced PYK2 expression [16], and that PYK2 enhances STAT3 phosphorylation on its primary activating tyrosine site (Y705) [16]. Together, these data established a transcriptional positive feedback loop between PYK2 and STAT3, which is described by reactions 5, 6, 9, 10 and 11 in the model’s reaction scheme (Fig 1A) and by F2 in Fig 1B. Further, we showed that EGF stimulation of MDA-MB-468 cells, as opposed of PYK2-depleted cells induced co-localization of EGFR with phospho-STAT3 (pY705) at endosomal-like structures [12], implying that PYK2 is essential for EGFR-STAT3 interaction. The association between PYK2, EGFR and STAT3 was further confirmed by co-immunoprecipitation studies [12]. Consequently, the EGFR-PYK2-STAT3 interactions are modelled as a sequential cascade of signalling activation, as shown in Fig 1A.

### Positive feedback between PYK2 and c-Met via STAT3

We previously showed that PYK2 positively regulates the steady-state level of c-Met protein as well as its transcription in response to EGF stimulation in TNBC cells [16]. We also showed that inhibition of c-Met using a specific inhibitor (PHA-665752) reduced the phosphorylation of both PYK2 and STAT3 in response to EGFR stimulation [16], while STAT3 inhibition decreased c-Met transcription, suggesting that STAT3 enhances the transcription of c-Met (reaction 13). Together, our model (i) suggests a positive feedback loop in the PYK2-STAT3-c-Met axis (F4 and F3 in Fig 1B) and (ii) incorporates the degradation of c-Met by Cbl-mediated ubiquitination [19] (reaction 16).

### Model implementation and calibration

#### Model inputs, outputs and formulation

Binding of HGF to c-Met induces c-Met phosphorylation of two tyrosine residues (Y1234 and Y1235) within the catalytic loop of the tyrosine kinase domain [20], and the subsequent recruitment of effector proteins including growth factor receptor bound protein 2 (GRB2) and Src homology-2-containing (SHC) [20] which leads to activation of the Ras-Raf-MEK-ERK signalling cascade [21]. To keep the model minimal without compromising its dynamic accuracy, we simplified this multi-step signalling cascade into a single lumped reaction where ERK phosphorylation and activation are catalysed by both EGFR and c-Met (reaction 23). We assumed EGF and HGF as inputs into the model, while activities of STAT3 and ERK indicated by their phosphorylated levels are considered as the main model outputs, which together regulate cancer cell proliferation and survival [12,16].

We integrated all the essential molecular interactions and feedback regulation described above, based on which an ODE model was formulated using a combination of kinetic laws including Michaelis-Menten (MM) kinetics for catalytic reactions (e.g. (de)phosphorylation, (de)ubiquitination), Hill kinetics (transcription) and mass-action kinetics (association/ dissociation) [22–24]. As a result, the model comprises 13 ODEs and 70 kinetic parameters. Full description of the model reactions, ODE equations and parameters is given in S1 & S2 Tables.

#### Model calibration and kinetic parameters estimation

The adequacy of a model is generally justified by its ability to recapitulate experimental data. Thus, to ensure model quality we calibrated (trained) the EGFR-PYK2-c-Met model using time-course and perturbation data obtained from TNBC MDA-MB-468 cells. These include the dynamic response of phosphorylated and total levels of key network components (EGFR, PYK2, c-Met, ERK and STAT3) following EGF stimulation (Fig 1C–1G). While EGF stimulation monotonically increased the activities of EGFR, ERK and STAT3, it instead induced a biphasic response of c-Met where c-Met level was initially decreased but then dramatically increased beyond its basal level (Fig 1G). In addition, time-course profiles of PYK2 and c-Met mRNA measured over 8 hours (Fig 1H–1J), and of pSTAT3, pERK1/2, c-Met and PYK2 measured over 2 hours (24 hours for c-Met) following EGF stimulation in control and PYK2-deficient cells (Fig 1K–1R) were used for model training. Finally, data regarding the effect of c-Met inhibition on signalling nodes were also used (S1A and S1B Fig).

Model calibration involved the estimation of unknown model parameters using a global parameter estimation method called genetic algorithm [22,25–27] implemented in Matlab and run in parallel on high-performance supercomputers at Monash University (see Methods for details). Model simulations using the optimal parameter values successfully recapitulated the observed network dynamics demonstrated by the strong agreement between model simulations and quantified data (Fig 1C–1T), as well as by a quantitative assessment (S2 Fig and S1 Text). The fitted model also accurately captured the intricate detail involving the biphasic dynamics of EGF-induced c-Met expression (Fig 1G). Moreover, additional independent simulations have further validated the model’s predictive capability. Specifically, simulation results of constitutive STAT3 activation qualitatively recapitulated the corresponding experiment data not used in training (S1C and S1D Fig). Together, these analyses demonstrate that the model is predictive and could be used to make new predictions.

### Identification and predictive ranking of synergistic drug combinations

While synergistic drug combinations have been identified for different cancers, most combinations rely on empirical observations or expensive drug or siRNA/shRNA/CRISPR based screens that lack mechanistic anchoring and scalability [28–31]. As our calibrated EGFR-cMET-PYK2 model recapitulated the experimental data, we asked whether it can identify and prioritize synergistic drug combinations.

**Fig 2:**
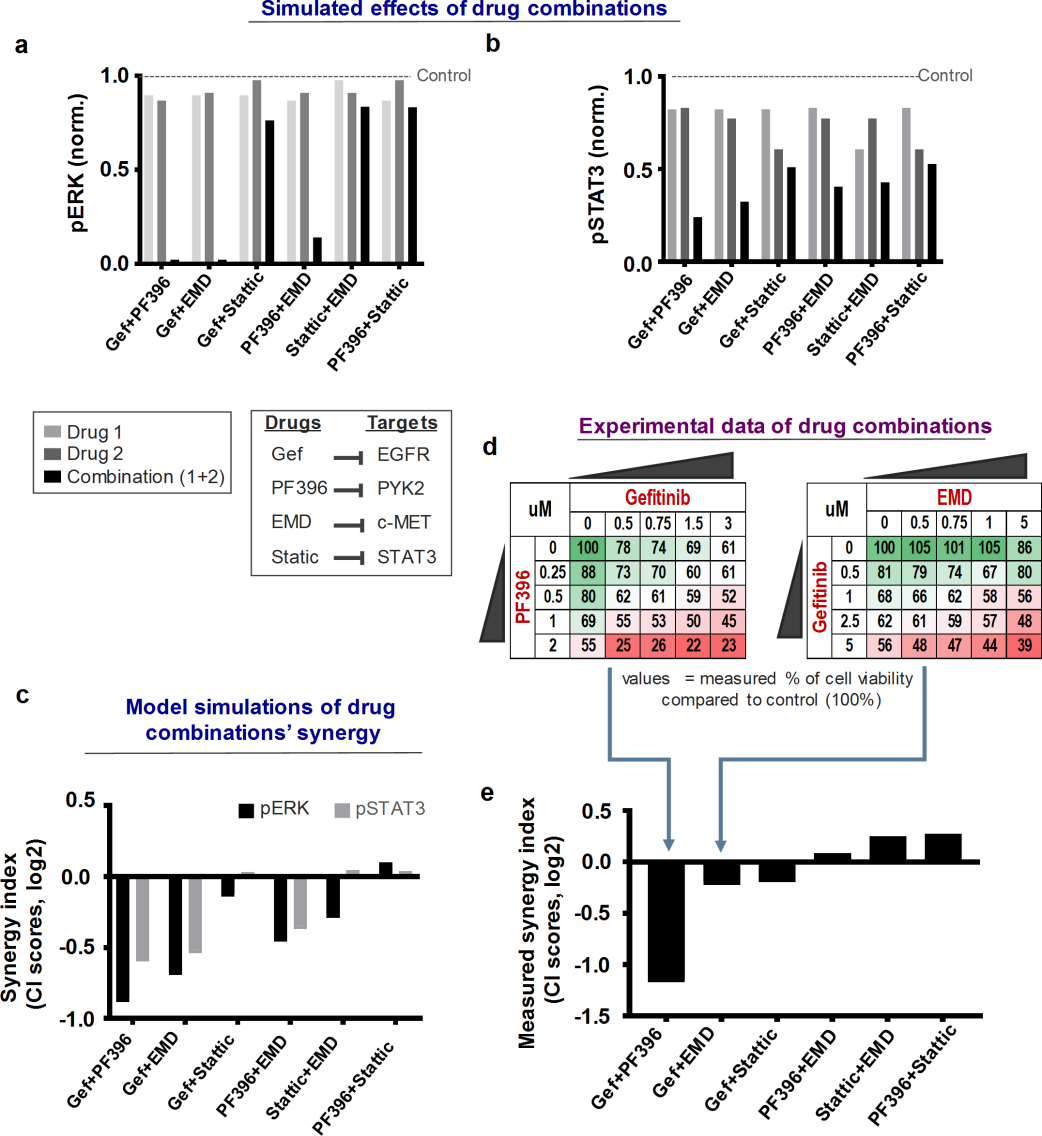
Model-based identification and prioritization of drug combinations. **(a-b)** Model simulations of the effects of single-drug versus combined-drug treatments for six drug combinations as indicated on pERK and pSTAT3 levels, which were normalised by total levels. D1: first drug, D2: second drug in the order of the x-axis’s label. IC_25_ concentrations were used for each drug. **(c)** Predicted level of synergy (synergy index, log2 scale) for each drug combination using the Chou-Talalay based CI scores (see main text and Materials & Methods for details). **(d)** Inhibition effects on cell (MDA-MB-468) growth by increasing combined levels of drug pairs (see also S8 Fig) compared to non-treated cells. Gradient colour code: from untreated (green) to strongly inhibited (red). **(e)** Drug combinations sorted by their corresponding synergy indices (CI scores), calculated based on the respective combination matrices in panel (d) and S8 Fig.

To this end, we considered EGFR, PYK2, c-Met and STAT3 as four potential targets with associated small-molecule inhibitors: Gefitinib, PF396, EMD and Stattic, respectively, and six different pair-wise combinations between them: Gefitinib+PF396, Gefitinib+Stattic, Gefitinib+EMD, PF396+EMD, PF396+Stattic and Stattic+EMD. To analyse the effects of these drug combinations on cell signalling, we simulated the steady-state levels of pERK and pSTAT3 in response to the individual drugs alone or in combinations, at their corresponding IC_25_ levels. IC_X_ is defined as the concentration at which a drug inhibits a signalling output (e.g. pERK/pSTAT3) by X% (X = 0–100) compared to the untreated condition (see S3 Fig). Model simulations showed that while single-drug treatments only modestly suppressed pERK/pSTAT3, the combined treatments produced greater-than-additive effects for co-inhibition of EGFR-PYK2 or EGFR-c-Met, while this is much less pronounced for co-inhibition of c-Met-STAT3 or PYK2-STAT3 (Fig 2A and 2B). Similar simulations were also observed when we varied the applied drug concentrations by using different IC values (S4 Fig), showing their consistency. Together, these simulations predicted varying levels of efficacy for different drug combinations, among which co-inhibition of EGFR and PYK2 and EGFR and c-Met seemed to yield effects significantly better than additive.

However, greater-than-additive effects do not necessarily indicate drug synergy. Thus, to systematically and quantitatively evaluate the level of synergism of the considered drug pairs, we employed different methods to calculate the levels of drug synergy (Chou-Talalay’s Combination Index, (CI score) [32], Bliss Independence (BI score) [33] and Coefficient of Drug Interaction (CDI score) [34], see Methods and S2 Text for details). Synergy scores <1, = 1 or >1 indicate synergistic, additive or antagonistic effects, respectively; with the magnitude of synergism/antagonism signified by how much smaller/larger they are compared to 1. As shown in Fig 2C, our model predicted a strong synergy between Gefitinib and PF396 or EMD, indicated by the CI scores much less than 1 for both pERK and pSTAT3 as outputs. In contrast, the CI scores for the combinations EMD-Stattic and PF396-Stattic are marginally different to 1 for both pERK and pSTAT3, indicating these drug pairs are unlikely to yield synergistic effects. In addition, co-targeting EGFR-STAT3 (by Gefitinib+Stattic) was predicted to be mostly additive, while co-targeting c-Met-PYK2 (by EMD+PF396) was slightly synergistic, which is more pronounced for pERK than pSTAT3. Importantly, these simulations were consistent between different synergy computational methods (see S6 and S7 Figs for BI and CDI scores). As pERK and pSTAT3 are major regulators of cell growth and survival, our simulations collectively led us to hypothesize that drug combinations that co-target EGFR-PYK2, EGFR-c-Met or PYK2-c-Met may yield synergistic effects in inhibiting TNBC growth, while those co-targeting STAT3 with either c-Met or PYK2 are unlikely to induce synergy.

To experimentally validate model predictions, we exposed TNBC MDA-MB-468 cells to increasing levels of Gefitinib, PF396, EMD, and Stattic in six combinations, and measured the effects on cell viability using the MTT assay and colony formation assay (Fig 2D and 2E and S8 Fig). To mitigate possible off-target effects (e.g. PF396 is known to also inhibit FAK), we aimed to use minimal drug concentrations sufficient to suppress downstream signalling. The combination index values (CI) were then calculated (CompuSyn software, CompuSyn. Inc) and synergistic (CI < 1), additive (CI = 1), and antagonistic (CI > 1) effects were defined.

As shown, the experimental data supported our predictions, and indicate that Gefitinib+PF396 was the most synergistic combination, followed by Gefitinib+EMD and Gefitinib+Stattic, whereas combined treatments of EMD+Stattic and PF396+Stattic did not show synergistic effects but mildly antagonistic (Fig 2D and 2E). Consistent with these results, we have recently showed that co-targeting of EGFR and PYK2 substantially reduced the growth of basal-like TNBC cells both in vitro and in animal models, and further showed that this drug combination is more potent than dual inhibition of EGFR and c-Met [12]. Taken together, these results suggest that our model not only accurately predicted synergistic drug combinations, but also correctly prioritized and ranked them according their efficacy, and thus, could be applied for selecting best drug combinations for clinical testing.

### Model-based analysis of dynamic network response to drug treatments

**Fig 3:**
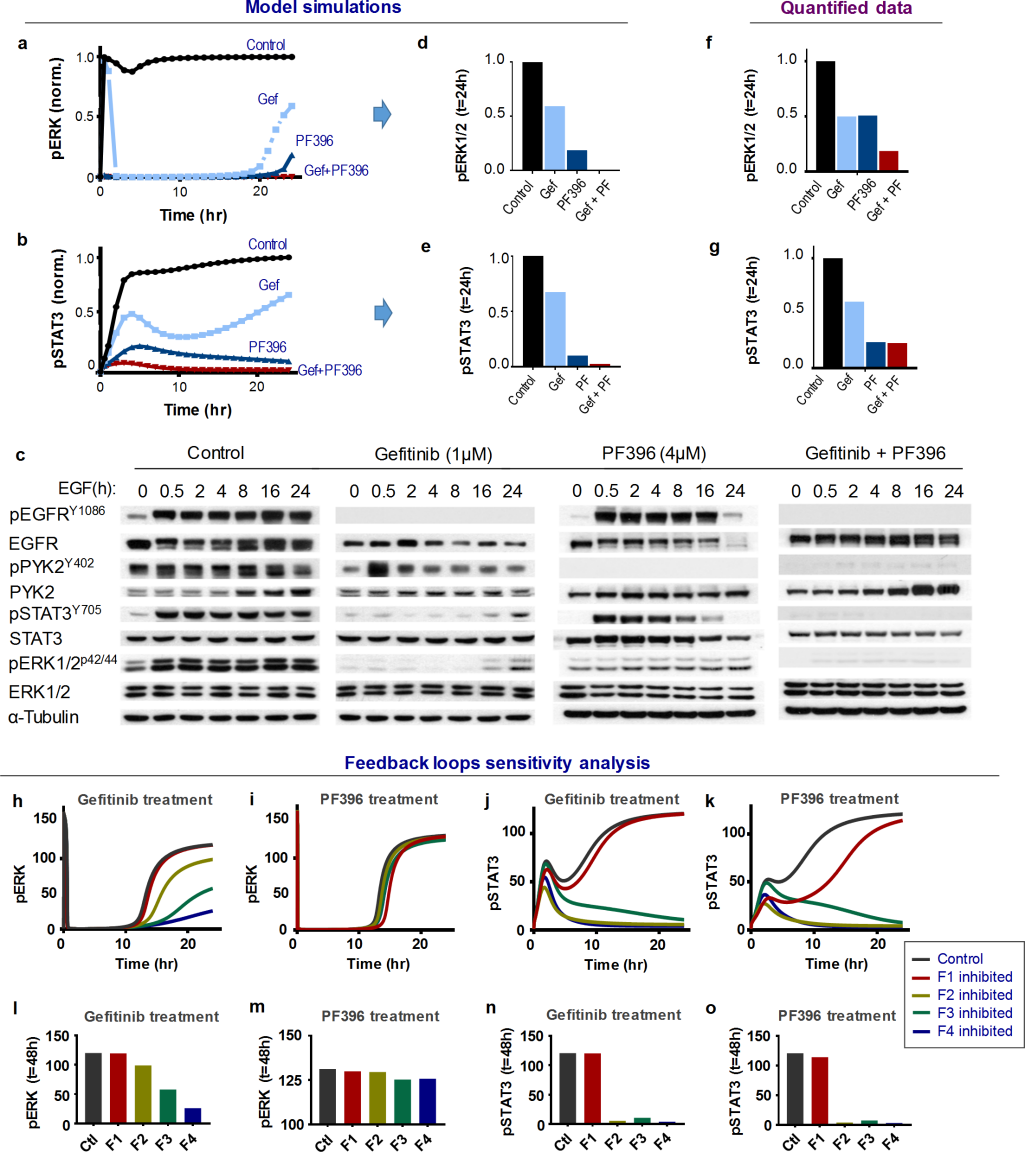
Analysis of time-dependent signalling pathway activation in response to dual inhibition of EGFR/PYK2. **(a-b)** Simulations of time-dependent phosphorylation of STAT3 and ERK in response to Gefitinib (Gef) and PF396 (PF) alone, or in combination. **(c)** Effects of Gefitinib and PF396 treatment on the protein levels and activation states of indicated signalling proteins upon indicated time-points of EGF stimulation. Shown are representative western blotting (WB) results. **(d-e)** Simulation data of steady-state pERK and pSTAT3 levels in response to single-drug and combined treatment of Gefitinib and PF396 for 24h. **(f-g)** Experimental data of steady-state pERK and pSTAT3 levels in response to single-drug and combined treatment of Gefitinib and PF396 for 24h. **(h-k)** Sensitivity analysis of the feedback regulation. Simulations of the effect of time-dependent feedback perturbation on pERK and pSTAT3 levels. F1-F4 indicates the feedback-related links perturbed for analysis (for detail see S4 Table). Each feedback-related link was alternatively perturbed by inhibiting its strength by 20%. **(l-o)** Steady-state levels of pERK and STAT3 following 48h of drug treatments comparing control and perturbations of the feedback loops.

Although the above drug combinations had synergistic effects, it was unclear why the steady-state activities of ERK and STAT3 were only marginally suppressed by single-drug treatments but markedly reduced by the combinations (Fig 2A and 2B). To investigate this we focused on EGFR-PYK2 co-targeting, and utilized the model to simulate the time-dependent signalling responses to EGF stimuli in the absence or presence of Gefitinib, PF396 or both. Interestingly, model simulations showed that although ERK activity was rapidly inhibited by Gefitinib as a single-drug treatment, the inhibition was transient and pERK bounced back at 24 hr (Fig 3A). The combined Gefitinib + PF396 treatment, however, abolished this late pERK recovery. Moreover, simulations showed that Gefitinib have similar bounce-back effects on pSTAT3, albeit the latency time caused by single-drug treatment (duration of brief suppression) were slightly different to that of pERK (Fig 3B). Similarly, model simulations suggest that single-drug treatment with PF396 alone did not efficiently suppress pERK/pSTAT3 compared to the combination, inducing a slight recovery of pERK at 24 hr and a transient response in pSTAT3 (Fig 3A and 3B).

To validate these predictions, we stimulated MDA-MB-468 cells with EGF in the absence or presence of Gefitinib, PF396 or both, and assessed the protein and phosphorylation levels of the key signalling nodes of the EGFR-c-Met-PYK2 network over 24 hours (Fig 3C, quantified in S10A and S10B Fig). Active EGFR and PYK2 phosphorylations were strongly inhibited under Gefitinib (1μM) and PF396 (4μM) treatment, respectively, indicating these drugs acted on the desired targets (see also S11 Fig). Consistent with model simulations, the experimental data indeed reproduced the predicted late bounce back of both pERK and pSTAT3 in response to Gefitinib treatment alone (Fig 3C, 2^nd^ column), but not to the combined treatment (Fig 3C, right column). The drug combination markedly outperformed both drugs as single-agent treatment in long-term suppression of ERK/STAT3 (Fig 3F and 3G), consistent with model predictions (Fig 3D and 3E). Moreover, PF396 treatment alone did not significantly affect ERK activity, and induced a transient response of pSTAT3 which is in accordance with previous simulations. Interestingly, further simulations predicted that the dynamic patterns of ERK and STAT response depend on PF396 dosage, with late bounce back of pERK appearing at lower PF396 concentration, accompanying a disappearance of the transient pSTAT3 response (S10C and S10D Fig). Treating MDA-MB-468 cells with a lower PF396 concentration indeed confirmed these predictions (S10E–S10G Fig). Together, these simulations and experimental data suggest that Gefitinib and PF396 as single agents failed to induce durable inhibition of ERK/STAT3 activation, while their co-treatment blocked the late bounce back of these oncogenic signals, and thus resulting in synergistic effects.

To assess the contribution of feedback loops to monotherapy treatment and the associated late response of pERK/pSTAT3 re-activation, we systematically perturbed each feedback loop (F1-F4) *in silico*. Interestingly, the late pERK response induced by Gefitinib was controlled by the PYK2-STAT3-c-Met positive feedback loop (F3/F4), while F1 and F2 had relatively minor influence (Fig 3H, quantified in Fig 3L). In contrast, feedback perturbations did not seem to significantly affect the magnitude of the PF396-induced ERK response as well as its latency time (Fig 3I and 3M). For pSTAT3 as a readout, F2/F3/F4 significantly regulate its late response caused by both Gefitinib and PF396, while F1 had no effect (Fig 3J and 3K, quantified in Fig 3N and 3O). These results suggest that EGFR inhibition in combination with interference of the PYK2-c-Met positive feedback loop (F3/F4) and thus PYK2 or c-Met inhibition, synergistically block ERK activation, as we predicted (Fig 2C) and supported by experimental data (Fig 2D and 2E).

Overall, these results demonstrate the power of our kinetic model in precisely predicting the time-dependent responses of network dynamics to drug treatments. The model-based analyses helped to identify the dynamic molecular features that underlie the re-activation of survival signals and to prioritize synergistic combinations.

### Switch-like signalling and phenotypic switches mediate drugs synergy

**Fig 4:**
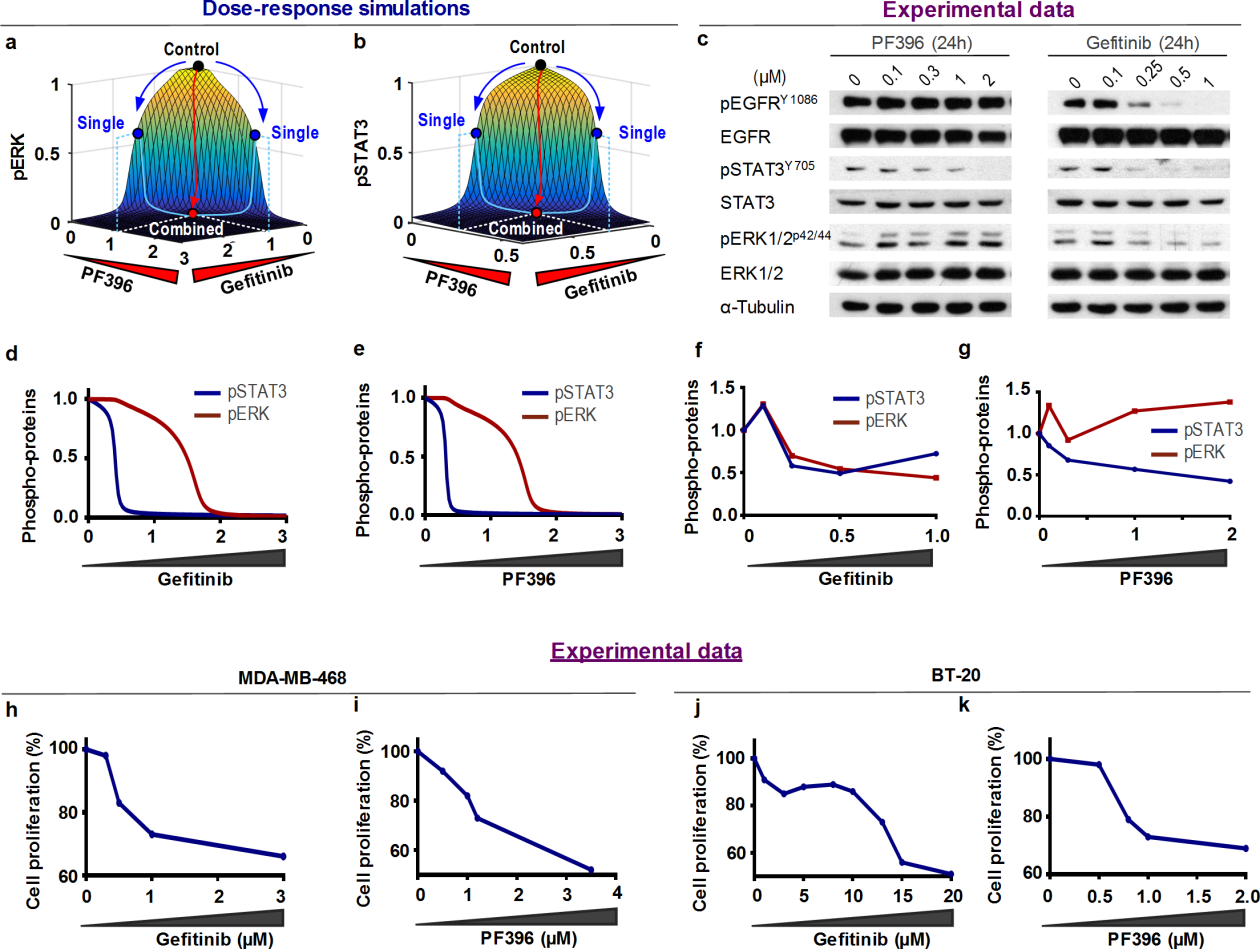
Drug-mediated switch-like responses are linked to drug synergy. **(a-b)** 3-dimensional (3D) simulations of the normalised steady-state pERK and pSTAT3 levels to simultaneous increases of Gefitinib and PF396 concentrations. The simulation shows a switch-like response pattern. Activation states of ERK and STAT3 were slightly suppressed upon single-drug treatments (blue dots), while the combined treatment significantly suppressed these activation states (red dots). **(c)** Experimental data of the effect of increasing concentrations of Gefitinib or PF396 for 24h on the protein levels and activation states of indicated signalling proteins. Shown are representative WB results of reproducible experiments of MDA-MB-468 cells. **(d-e)** 2D dose-response simulations of steady-state pERK and pSTAT3 levels to Gefitinib and PF396, respectively. **(f-g)** Quantification of experimental data in **(c)**. **(h-k)** Cell proliferation dose-response curves of Gefitinib or PF396 treatment assessed by MTT assay in MDA-MB-468 and BT-20.

Simulations of our calibrated model showed that pERK/pSTAT3 responded in a switch-like manner to increasing concentrations of EGF or HGF (S9 Fig). As switch-like responses are often emerged from a combination of positive and negative feedback regulation [35,36], we examined the possible link between the degree of switch-like response and drugs synergy in our model system. Simulation of the 3-dimensional (3D) dose-responses of pERK/STAT3 to increasing concentrations of individual drugs using the six drug pairs applied earlier (Fig 4A and 4B and S12 Fig), clearly showed a highly switch-like response landscapes of both pERK and pSTAT3 to concurrent changes of Gefitinib and PF396 (Fig 4A and 4B). As shown, each individual drug (blue dot) slightly inhibited the signal outputs compared to control (black dot), while intermediate doses of the combined drugs dramatically pushed the output toward the bottom of this steep landscape (red dot), generating a cooperative effect. Importantly, the three other predicted synergistic combinations: Gefitinib + PF396, Gefitinib + EMD and Gefitinib + Stattic, but not the non-synergistic combinations (PF396 + EMD, Stattic + EMD, PF396 + Stattic; S12 Fig), also displayed switch-like responses (Fig 4A and 4B and S12 Fig) for the two signalling output pERK and pSTAT3. These analyses reveal a link between switch-like behaviour of drug responses and drug cooperativity.

Importantly, we confirmed these switch-like responses also by 2D dose-response simulations of Gefitinib and PF396 (Fig 4D and 4E). In addition, these simulations suggest that pSTAT3 is more sensitive to both drugs than pERK, indicated by a shift to the left of its dose-response curves (blue vs red, Fig 4D and 4E). To verify these predictions, we exposed MDA-MB-468 (Fig 4C and 4H and 4I) and BT-20 (Fig 4J and 4K) cells to increasing concentrations of either Gefitinib or PF396 and assessed their impact on ERK and STAT3 phosphorylation (Fig 4C) and on cell growth (Fig 4H–4K). As shown, pERK and pSTAT3 responded in a switch-like fashion to increasing Gefitinib, being high at low doses but jumping to significantly lower levels at higher doses of Gefitinib (Fig 4F). pSTAT3 was more sensitive to both drugs compared to pERK as correctly predicted (Fig 4D and 4E). Furthermore, the switches detected at the signalling level were also translated into switch-like behaviour at the cell growth level in both TNBC cell lines; further demonstrating the power of our predicted modelling (Fig 4H and 4J) [12].

Further model simulations predicted that Gefitinib can sensitize the cells to PF396 and vice versa, as evident by a left shift in the dose-response curves and the lower IC50 values (S13A–S13C Fig and S13G–S13I Fig). These predictions were confirmed by experimental data demonstrating that Gefitinib treatment indeed sensitized TNBC cells to PF396 (S13D and S13E Fig, red vs. blue curves), and reduced the IC50 of PF396 in both cell lines (S13F Fig). Similarly, knockdown of PYK2 by shRNA markedly sensitized MDA-MB-468 and BT-20 to Gefitinib (S13J–S13L Fig).

Together, our combined analysis revealed a switch-like pattern as a feature of the steady-state response profiles of the EGFR-PYK2-c-Met network, and further demonstrated the link between cell proliferation-associated switch-like response and drug synergy, implying that a similar response pattern in other signalling networks could be used to predict drugs synergy.

### Analysis of feedback loops underlying switch-like behaviours

**Fig 5:**
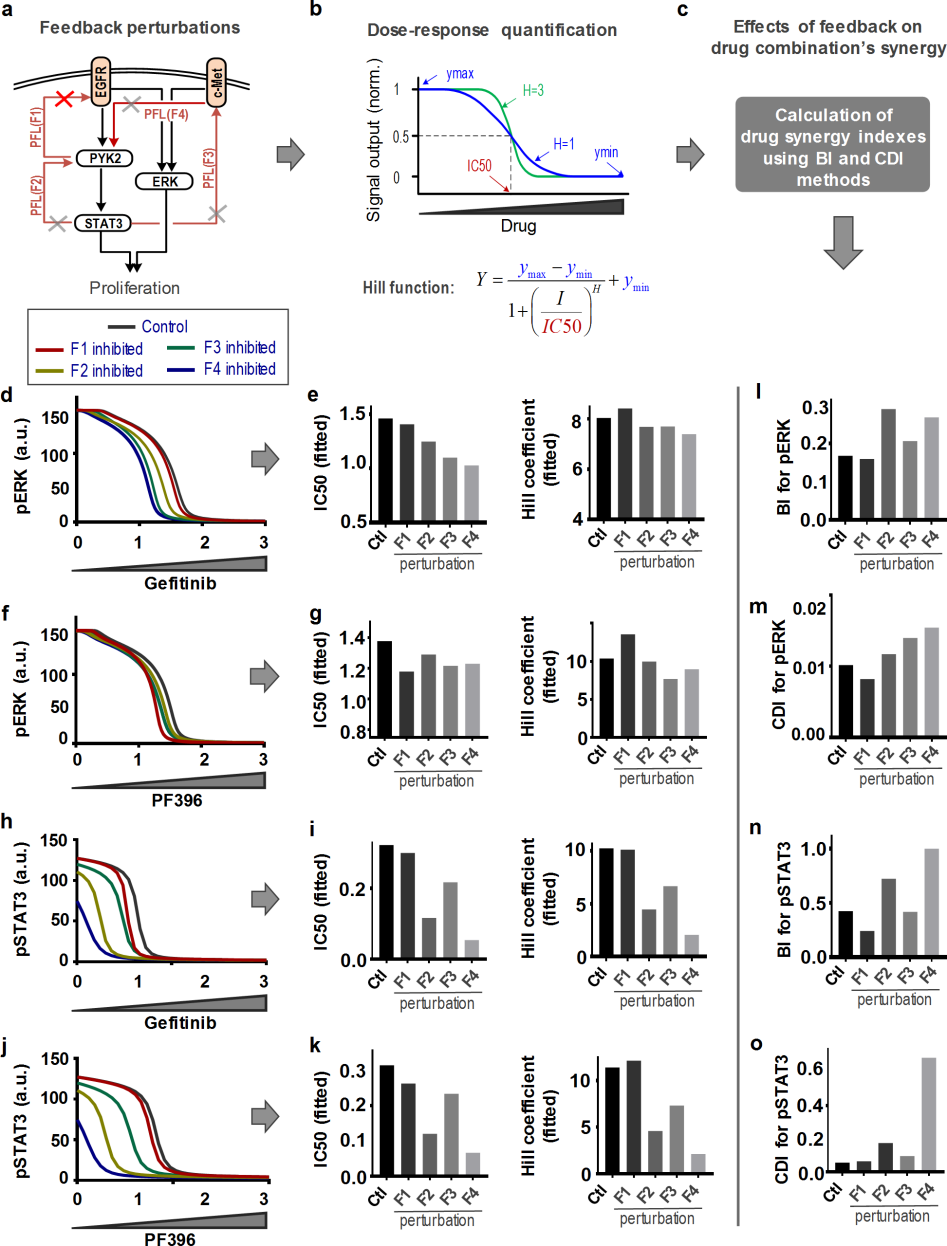
Analysis of feedback loops underlying switch-like behaviours. **(a)** Feedback perturbation of the EGFR-PYK2-c-MET network model. **(b,c)** Characterization of dose-response curve and synergy indexes (see also S14 Fig)**. (d)** Simulated dose-response profiles of pERK levels to Gefitinib. **(e)** IC50 and Hill coefficient values estimated from the dose-response curves of (d), where the strength of each feedback link was inhibited by 20%. **(f)** Simulated dose-response profiles of pERK levels to PF396. **(g)** IC50 and Hill coefficient values estimated from the dose-response curves of (f). **(h)** Simulated dose-response profiles of pSTAT3 levels to Gefitinib. **(i)** IC50 and Hill coefficient values estimated from the dose-response curves of (h). **(j)** Simulated dose-response profiles of pSTAT3 levels to PF396. **(k)** IC50 and Hill coefficient values estimated from the dose-response curves of (j). **(l-o)** Simulated effects of feedback perturbations on drug synergy for (d-g) pERK and (h-k) pSTAT3, indicated by the BI and CDI values. F1-F4 indicates the feedback-related links perturbed for analysis (for detail see S4 Table).

Although positive feedback is known to drive switch-like responses in biological systems [35,36], the fact that the EGFR-PYK2-c-Met network is governed by several positive feedback mechanisms (F1-4) led us to seek for the predominant feedback(s) in drug-induced switch-like features via systematic feedback perturbation analysis (Fig 5A), focusing on the Gefitinib+PF396 drug combination.

Switch-like dose-response curves can be quantitatively characterized by fitting the curves to a Hill function where the Hill coefficient and IC50 value signify the curve steepness (sigmoidality) and the sensitivity to the inhibiting drugs, respectively (Fig 5B and S14 Fig) [37,38] (see Methods for details). Interestingly, proportionally equal perturbations of the feedback loops did not result in equivalent changes in the dose-response profiles. F4 exerted strongest control as its disruption significantly shifted these curves to the left with reduced sigmoidality, as evidenced by lower IC50 and Hill coefficient values, particularly for pSTAT3 as output (Fig 5D–5K). This suggests F4 strongly promotes the switch-like response of STAT3 to both Gefitinib and PF396. Conversely, F1 was found to have little impact on all dose-response curves, indicating this feedback plays a minor role in shaping these switches. In addition, we found the pSTAT3 switches are also sensitive to F2 blockage, while the pERK switches are slightly sensitive to F3 disruption instead.

Next, we asked if these feedback links influence the synergy between Gefitinib and PF396. Thus we re-evaluated the synergy indexes (BI and CDI values) with respect to pERK and pSTAT3 under similar feedback perturbations. Shown in Fig 5L–5O, disruption of F2 and particularly F4 dramatically increased the BI/CDI values for both signalling outputs, suggesting these links strongly promote the drugs’ synergistic effect. Together, these results reveal F4 as a key feedback link that promotes both switch-like behaviour and drug synergy, further supporting a connection between these entities.

### Model-based stratification of patients identifies subgroups benefiting from combinatorial therapies

**Fig 6:**
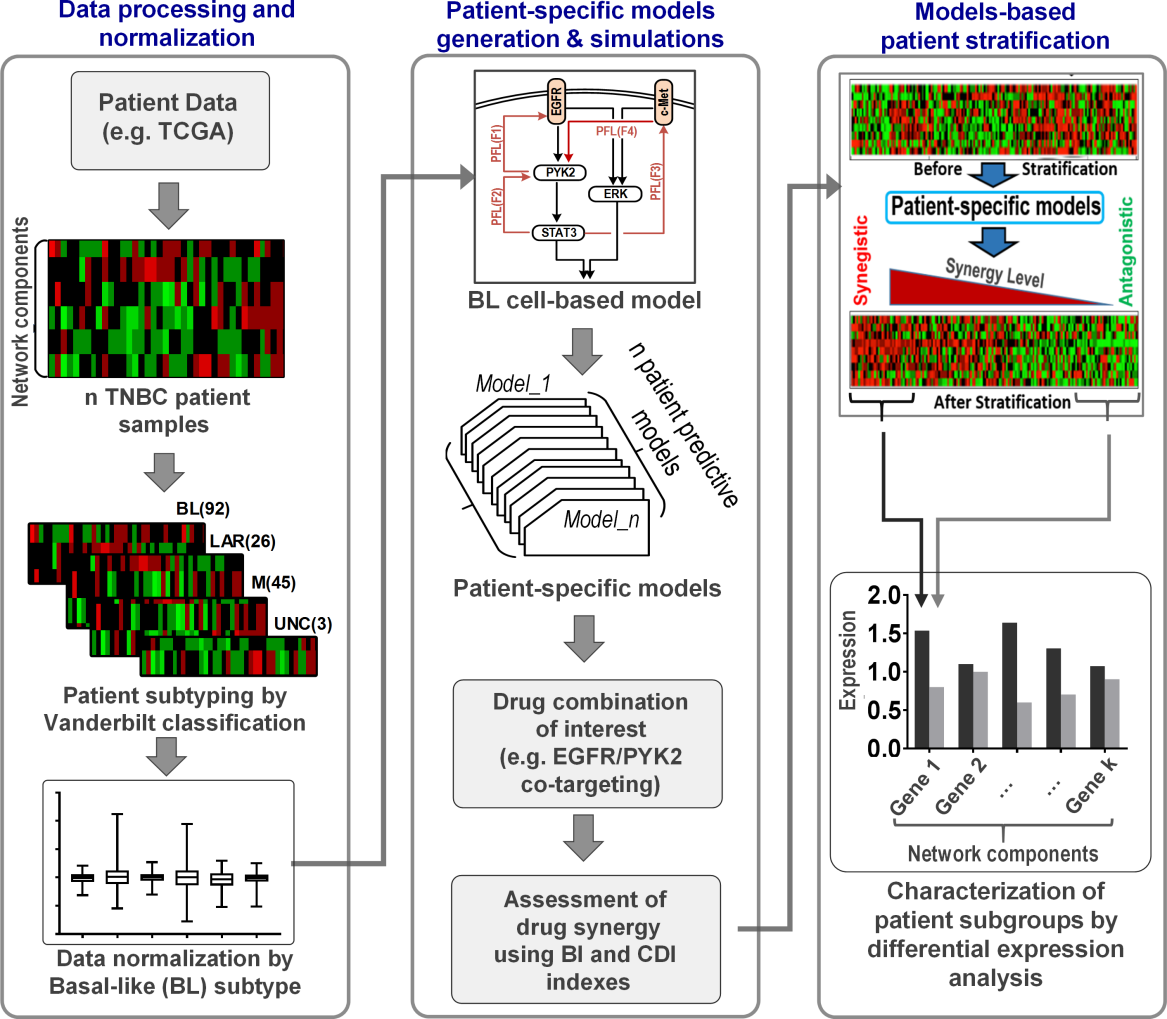
A computational analysis pipeline for patient stratification. The pipeline consists of 3 phases. TNBC patient samples (e.g. from TCGA) are subclassified into subgroups based on Vanderbilt subtyping (left panel). The sample data is normalized by the median value of the BL subtype (see text for details), which is then incorporated in the network model to generate patient-specific network models (middle panel). Simulations using patient specific models predict synergic effect by Gefitinib plus PF396 co-treatment. Patients are sorted according to the predicted synergy (right panel). Subgroups comprising highly synergistic (Q1) and non-synergistic patients (Q2) (i.e., low and high BI/CDI) are identified. Differential expression analysis is conducted to identify molecular patterns (biomarkers) that characterize these subgroups.

We next exploited our model to predictively stratify TNBC patients into subgroups who may or may not benefit from a certain drug combination, focusing on the EGFR-PYK2 co-inhibition as an example. To this end, we developed a general computational pipeline to generate patient-specific models using patient data, and compute the level of predicted synergy towards a chosen drug combination in each patient using their corresponding model (Fig 6). Sorting the patients by the predicted synergy levels allows us to group them into subgroups displaying different levels of response to the combination, thereby stratifying the patients. Furthermore, differential expression analysis across these subgroups enables the identification of potential molecular patterns that differentially characterize these subgroups (Fig 6, right).

We first obtained protein and gene expression data from a TNBC cohort (called the ‘discovery cohort’) containing 108 patients from The Cancer Genome Atlas project [39] (see Materials & Methods for details). Using the Vanderbilt-based subtyping system [40], the patients were classified into different subgroups: Basal-Like (BL1/2), Mesenchymal (M), Luminal Androgen Receptor (LAR), and unclassified (UNC). Examining the mutation profiles of these patients showed that the patient heterogeneity primarily comes from variation in expression levels of the network nodes rather than point mutation events (S6 Table). As our model was constructed based on the MDA-MB-468 cell line previously aligned to the Basal-Like (BL1) TNBC subtype (18), expression data from each patient was normalised by the median values of the BL1 patient subgroup (Fig 6, left). This normalisation step allowed us to tailor the cell-based model for each patient by adjusting the expression levels of the model species according to the normalised fold change. For instance, if a patient displays a two-fold increase in EGFR expression, then the EGFR abundance is doubled in the model describing that patient. This procedure was followed for all the model components resulting in 108 personalized kinetic models of the EGFR-PYK2-c-Met network (see Materials & Methods for more details). Note that an alternative normalisation strategy where we normalised all the patients against a patient whose expression profile most closely resembles that of the MDA-MB-468 cell line did not change the results’ main conclusions (S18 and S19 Figs). Using these patient-specific models, we next computed the predicted synergy levels of the Gefitinib+PF396 combination using the BI scores for each patient, which were then used to rank the patients, allowing identification of patient subgroups with different susceptibility to the co-treatment. To enhance the prediction accuracy of drug synergy, we excluded patients who did not respond at all to either drug when treated alone as informed by simulations.

**Fig 7:**
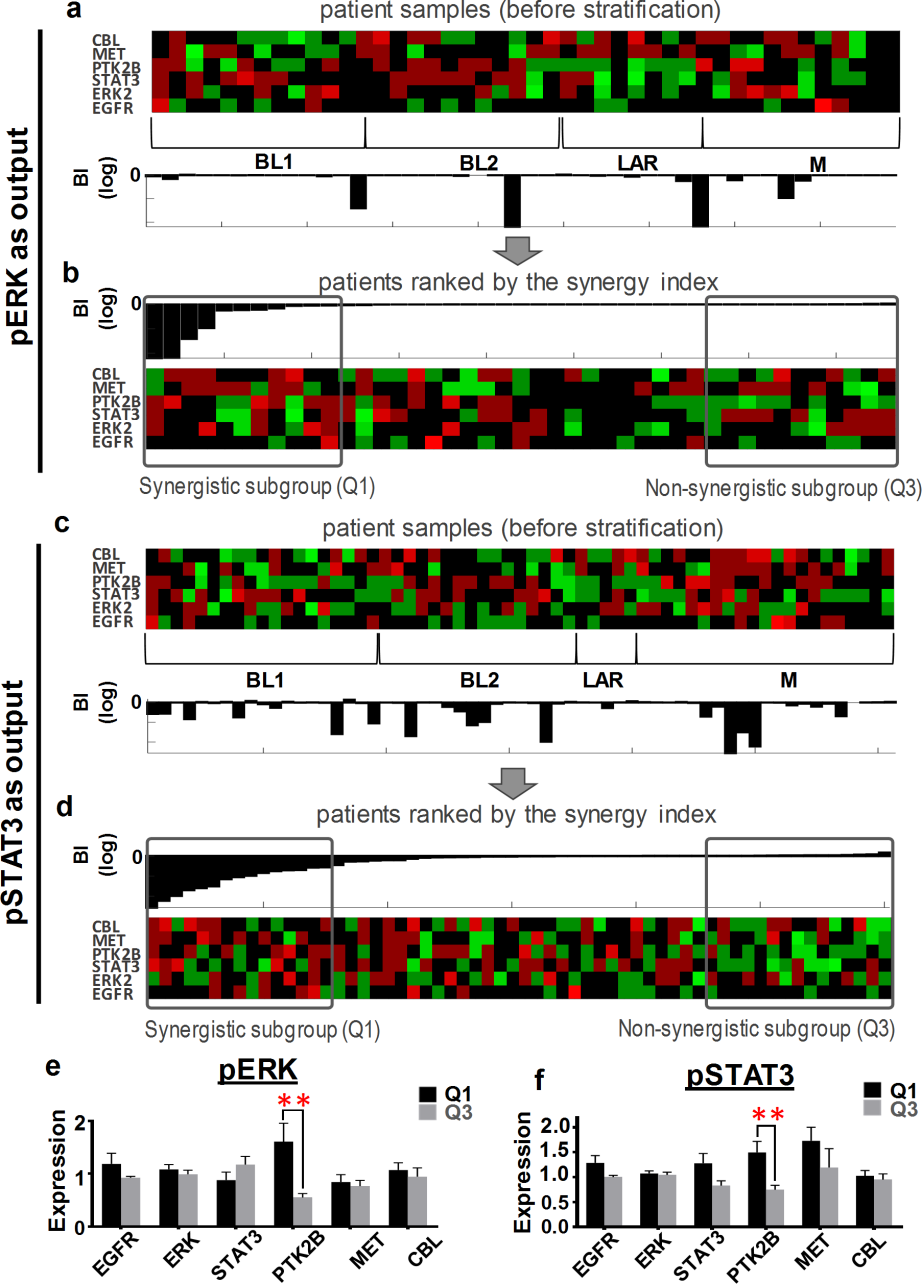
Identification of gene expression patterns. TNBC samples from the discovery cohort were clustered into Vanderbilt-based subtypes (upper panel). Synergy indices (BI) of EGFR and PYK2 co-inhibition predicted for each patient using the patient- specific network model (lower panels) are depicted. Only patients displaying sensitivity to Gefitinib and PF396 alone are included for synergy analysis (i.e. 44 and 61 patients for pERK and pSTAT3 as readouts, respectively). IC50 concentrations were used for each drug. **(a-b)** Stratification analysis with pERK as readout. Patient samples sorted by the predicted synergistic effect. Q1 and Q3 denote the ‘synergistic’ and ‘antagonistic’ patient subgroups, respectively. Q1 and Q3 contain patients having synergy index values in the lower quartile and in the upper quartile, respectively. **(c-d)** Stratification analysis with pSTAT3 as readout as in panels (a-b). **(e-f)** Differential gene expression analysis between the Q1 and Q3 subgroups, showing upregulation of PYK2 is statistically significant in Q1 subgroup for both signalling readouts. *P<0.05; **P<0.01.

As shown in Fig 7, the network nodes exhibit highly heterogeneous expression patterns across different TNBC subtypes within the discovery cohort (Fig 7A and 7C, top, and S18 Fig). Interestingly, computation of the drug synergy index showed that patients displaying potent or weak synergistic effects towards ERK (or STAT3) inhibition were found in all subtypes (Fig 7A and 7C, bottom & S19 Fig). Moreover, the synergy index did not show any significant statistical differences between the subtypes (S16 Fig), suggesting classical TNBC subtypes may not be sufficient to predict the synergy of the Gefitinib+PF396 co-treatment. Next, we sorted the patients by the synergy index (Fig 7B for pERK as output and Fig 7D for pSTAT3) and identified a highly ‘synergistic subgroup’ (termed Q1) and a ‘non-synergistic subgroup’ (Q3), containing patients having the synergy index values < the lower quartile and > the upper quartile, respectively. Differential expression analysis revealed that PYK2 was significantly upregulated in Q1 compared to Q3, which were consistent for both pERK and pSTAT3 as outputs (Fig 7E and 7F). To validate these findings, we additionally performed the patient stratification analysis on a second, independent patient cohort (‘validation cohort’, described in Materials & Methods). Consistent with the discovery cohort, model simulations predicted PYK2 is significantly upregulated in the synergistic patient group (S17 Fig). Taken together, these results suggest that upregulation of PYK2 could serve as indicators of susceptibility to treatments co-targeting EGFR-PYK2.

## Discussion

Kinases are the largest class of cancer therapeutic targets, yet most tumours can escape from the inhibition of any single kinase [14,15]. Although combination therapies targeting multiple kinases have become necessary to effectively combat cancer, it remains a major challenge to identify the best combinations and most susceptible patients for clinical testing. However, drug combination identification based on experimental screening techniques remains costly and often identify only small numbers of synergistic combinations, which motivates development of alternative computational approaches for the task. Notably, an open challenge was recently launched by the DREAM consortium to computationally predict pair-wise drug synergy in human diffuse large B-cell lymphoma [41], resulting in over 30 community-generated algorithms, several of which performed significantly better than random guessing. Most were statistical methods but an ODE-based kinetic modeling approach was also proposed [41]. While the accuracy of the predictions were suboptimal, these efforts strongly demonstrate that computational prediction of drug synergy is feasible and can be useful in advancing the development of effective therapeutic strategies. Here, we address the same challenge by employing a systems approach that integrates mechanistic kinetic modelling with experimental validation in an iterative manner. Focusing on the EGFR-PYK2-c-Met interaction network in TNBC as a model system, we demonstrated the significant potential of this integrative approach in the rational identification and prioritization of synergistic drug combinations, as well as predictive stratification of patients for optimal benefit from these combinations. The strength of this approach further lies in its flexibility and scalability where the presented principles and computational frameworks could be reused and adapted to investigate similar questions in other systems and/or other tumour types beyond TNBC.

Model predictions validated by in vitro experimental data showed that EGFR-PYK2 co-targeting yields most synergistic effect in basal-like TNBC cell lines among other possible combinations, followed by EGFR-c-Met co-targeting. Indeed, previous studies suggest that combined targeting of both EGFR and c-Met receptors could be an effective therapeutic strategy for TNBC [42], and have reported in vivo data supporting synergy in co-inhibition of EGFR-c-Met [43–45]. In addition, our recent results further demonstrated the potent synergistic effect of EGFR and PYK2/FAK co-targeting on basal-like TNBC [12]. While these experimental data were obtained both *in vitro* and *in vivo* using TNBC cell lines and xenograft mouse models, respectively, the systems approach described here, using a quantitative modelling of EGFR-PYK2-c-Met interaction network in TNBC, precisely predicted the outcome results and the obtained synergistic effects of the combined treatments. Not only our model-based analysis could identify highly synergistic target combinations, we also uncovered non-synergistic (antagonistic) combinations for TNBC, such as c-Met-STAT3 or PYK2-STAT3 co-targeting, that is also valuable in informing subsequent studies. While EGFR-PYK2 co-targeting was identified to be synergistic in TNBC, PYK2 may have a larger role in coordinating EGFR and c-MET signalling depending on the cancer types, evident by data showing PYK2 activation was not affected by Gefitinib in lung cancer cells [46]. Thus, the presence of drug synergy may be context specific and therefore should be treated accordingly.

Although effective drug combinations are being identified in many cancers, most resulted from empirical observations and/or expensive experimental screens [28–31] that frequently lack mechanistic anchoring, making it difficult to predict if the identified combinations may work in different biological contexts (and so patients). This issue is similarly faced by machine-learning or statistical based computational approaches aimed to predict effective drug combinations [47–49]. In contrast, our modelling captures the mechanistic details contained within the interaction network under study. Thus, it allowed us to link the molecular and dynamic network features with observed drug synergy. Indeed, time-dependent simulations backed up by experiments helped reveal that while inhibition of EGFR or PYK2 alone (by Gefitinib or PF396, respectively) only temporarily suppressed the activities of ERK/STAT3 which were subsequently restored to basal levels; this activity recovery was abolished by the combined treatment. This finding suggests that knowledge of drug synergy could be gained by understanding and exploiting the dynamic behaviour of signalling networks. We further found that the signalling and phenotypic switches mediated primarily by the positive feedbacks within the EGFR-PYK2-c-Met network contributed to the synergistic effects between Gefitinib and PF396. Importantly, feedback perturbation analysis unveiled that the PYK2-controlled feedback loops play crucial roles in mediating the signal restoration of ERK and STAT3. This analysis also helped pinpoint the PYK2-c-Met positive feedback loops as a critical regulator of the switches as well as drug synergy. These findings highlight the utilities of our mechanistic model in elucidating a systems-level understanding of drug-induced network dynamics and mechanisms that underlie monotherapy resistance as well as synergistic effects by drug combination.

The heterogeneous nature of TNBC is thought to underlie the sporadic and low response rates to targeted agents [50,51]. However, effective strategy in order to improve patient stratification is strongly lacking. While the expression or mutation status of the drug targets can inform drug response, these are not always sufficient [52] particularly in the context of multi-target inhibition, possibly because these biomarkers do not capture the mechanistic connection between the targets. We provided a proof-of-concept demonstration of the prognostic significance of our modelling by showing its ability in stratifying patients into subgroups exhibiting different susceptibility for a certain combination treatment through generation of patient-specific models utilizing patient-specific data. As an example, this enabled prediction of two patient subgroups, one highly susceptible to EGFR-PYK2 co-targeting and one least likely to benefit from the same combination. Importantly, differential expression analysis revealed upregulation of PYK2 was particularly enriched in the highly responsive subgroup, consistent between two independent patient cohorts analysed. Interestingly, upregulation of EGFR was not predicted to be significantly enriched in this group, however this finding was not surprising given that high EGFR expression is not always predictive of sensitivity to EGFR-directed inhibition [53]. These results support the notion that network-based biomarkers, rather than expression of the drug target, could offer better sensitivity indicator for drugs co-targeting. While the patient stratification analyses here were mainly focused on the EGFR-PYK2 co-inhibition, the patient-specific models could be utilised to discover new drug combinations in each patient for personalized therapies. Moreover, as our models were primarily kinases based, future availability of high-resolution proteomic data from patients instead of transcriptomic data would potentially further enhance model predictability.

In summary, our systems modelling presents a powerful and highly adaptable approach for the rational design and predictive prioritization of combinatorial therapies in an individualized manner, providing a concrete step towards personalized treatment for TNBC and other cancers. Given the space of possible drug combinations is vast while clinical trials are slow and costly, this predictive approach opens exciting avenues for drug repurposing and quick translation of combination therapies into the clinic.

## Methods

### Antibodies, reagents and chemicals

The following antibodies were purchased from Santa Cruz Biotechnology (Santa Cruz, CA, USA): rabbit polyclonal antibodies against pPYK2 (Y402, sc-101790, 1:1,000 WB), STAT3 (C-20, 1:1,000 WB), ERK1/2 (sc-93, 1:1,000 WB), pERK1/2 (pT202/ pY204.22A, sc-136521, 1:500 WB), cMet (sc-10, 1:1000 WB) and mouse monoclonal antibody against pSTAT3 (Y705, sc-8059, 1:500 WB). Mouse monoclonal antibody against α-Tubulin (T6074, 1:10,000 WB) was purchased from Sigma-Aldrich Israel (Rehovot, Israel). Antibodies against pc-Met (Y1234/5, #3077, 1:1,000 WB) and pEGFR (Y1068, #2234, 1:1,000 WB) were purchased from Cell Signalling Technologies (Danvers, MA, USA). Monoclonal antibody against EGFR (clone 111.6, 1:5,000 WB) was kindly provided by Prof. Y. Yarden (Weizmann Institute of Science, Rehovot, Israel). Polyclonal anti-PYK2 (1:500 WB) antibody was prepared as described previously [54]. Horseradish peroxidase-conjugated goat anti-rabbit and goat anti-mouse IgGs were purchased from Jackson ImmunoResearch Laboratories (West Grove, PA, USA). Gefitinib (G-4408) was purchased from LC Laboratories (Woburn, MA, USA). EMD1214063 (A3388) was purchased from Apexbio Technology (Houston, TX, USA). Stattic was purchased from Calbiochem (San Diego, CA, USA) and EGF, MTT, PF431396 (PZ0185) and other chemicals from Sigma-Aldrich Israel (Rehovot, Israel).

### Cell culture

MDA-MB-468 and BT-20 cells were obtained from ATCC (Manassas, VA, USA, 2013–2015). MDA-MB-468 cells were grown in RPMI 1640 (Gibco BRL; Grand Island, NY, US). BT-20 cells were grown in Eagle’s Minimum Essential Medium (MEM-Eagle’s) supplemented with 1 mM sodium pyruvate and 2 mM L-glutamine. All media preparations were supplemented with a penicillin–streptomycin mixture (100 U ml^−1^; 0.1 mg ml^−1^; Beit Haemek, Israel) and 10% fetal bovine serum (Gibco BRL, Grand Island, NY, US). Cell lines were verified to be mycoplasma negative. The two cell lines’ identities were verified by the provider by STR profiling every 6 months. Cells were expanded and subsequently stored in liquid nitrogen upon purchase. Original vials have only been passaged for not more than two months.

### Induction experiments and western blot analysis

To induce downstream signalling of EGFR, cells were stimulated with EGF (100 ng ml^−1^) for 30 min–24 h upon serum starvation (0.5% FCS) for 24 h. Phospho- and total protein levels were examined by western blotting (WB) which is described elsewhere.

### Dose response experiments

To investigate the changes in downstream signalling upon kinase inhibition cells were treated with several concentrations of inhibitor for 24 h and analysed by WB.

### IC_50_ values and drug synergism

IC_50_ values and drug synergism experiments were performed as described before [55]. In short, cells were seeded in 96-well plates and treated with increasing concentrations of one inhibitor for determination of IC_50_ values or in a dose matrix for determining the synergy indices (based on CI score using the Chou-Talalay method) for drug combinations. 72h after treatment cell viability was measured by MTT. IC_50_ values were calculated using GraphPad Prism version v.5.0 (GraphPad Software, California, USA) and CI values by CompuSyn software (CompuSyn.Inc, Paramus, NJ, US).

### Cell viability

Cell viability was assessed by MTT (3-(4,5-dimethylthiazolyl-2)-2,5-diphenyltetrazolium bromide) colorimetric assay as described previously [55]. In brief, cells were incubated in the absence or presence of the indicated drugs for 24–72 h as indicated. The cells were then incubated with medium containing MTT reagent (0.5 mg ml^−1^; Sigma) for 2 h at 37°C and then lysed in lysis solution containing 0.4% NP-40 in 0.04 mol l^−1^ HCl-isopropanol. Absorbance was measured at 570 nm and at 620 nm (reference wavelength), and cell viability was depicted as percentage of untreated control cells. Data is represented as the mean values of three independent biological replicates.

### Western blotting

Western blot analysis was performed as described previously [56,57]. Briefly, cells were extracted in cold lysis buffer (0.1% Triton-X-100, 50 mM Hepes pH 7.5, 100 mM NaCl, 1 mM MgCl_2_, 50 mM NaF, 0.5 mM NaVO_3_, 20 mM β-glycerophosphate, 1 mM phenylmethylsulphonyl fluoride, 10 μg ml^−1^ leupeptin, 10 μg ml^−1^ aprotinin) and centrifuged at 14,000 r.p.m. for 15 min at 4°C. Protein concentration was assessed by Bradford assay (Bio-Rad, Hercules, CA) and equal protein amounts (30 μg) were analysed by WB following SDS–polyacrylamide gel electrophoresis. ImageJ software (NIH, USA) was used to perform densitometric analysis.

### Mathematical model implementation, calibration and numerical simulation

#### Implementation

The mathematical model was formulated using ordinary differential equations (ODEs) and implemented in MATLAB (The MathWorks. Inc. 2016b). The model is also available in the standard SMBL format as part of this paper. All model simulations and analyses were performed using MATLAB. The ODE system was integrated and solved using the *ode15s* function in MATLAB, which is a variable-step and variable-order solver based on the numerical differentiation formulas (NDFs), and is specially designed for stiff systems.

#### Calibration

MATLAB’s Global Optimization toolbox was employed for model calibration and parameter estimation (see S1 Text for details). Specifically, a genetic algorithm [27] was used to find the optimal model parameter values that minimize the discrepancy between model simulations and the training datasets, by minimizing the following aggregated objective function:

$$M(p)=J(p)+\sum_{i=1}^{3}R_{i}(p),$$ (1)

where $J(p)=\sum_{j=1}^{m}{\sum_{i=1}^{m}\left(\frac{y_{j,i}^{D}-y_{j}(t_{i},p)}{\sigma_{j,i}}\right)}^{2}$ is the cost function (detail given in **S1 Text**); and *R*_*i*_(p)s represent the algebraic constraints described in the S5 Table.

Genetic algorithms are powerful methods to solve both constrained and unconstrained optimization problems based on a natural selection process that mimics biological evolution [58,59]. The algorithm repeatedly modifies a population of individual solutions. At each step, the genetic algorithm creates new individuals using crossover and mutation as well as selection of best individuals from the current population, and uses them as parents to produce the offspring for the next generation. Over successive generations, the population ‘evolves’ toward an optimal solution. Model fitting (parameter estimation) was performed using high-performance computing facilities at Monash University (http://www.monash.edu), consisting of two Haswell CPU sockets with a total of 16 physical cores (or 32 hyperthreaded cores) at 3.20 GHz and 300 TB usable storage.

#### Patient-specific models

Detailed description of patient-specific model generation is given in S3 Text. Patient-derived expression data (combining RPPA and RNA-seq based expression data) from The Cancer Genome Atlas (TCGA) was used as inputs to tailor the cell-based model for each patient and generate the patient-specific models. For instance, if a patient displays a two-fold increase in EGFR expression, then the EGFR abundance is doubled in the model describing that patient. This procedure was followed for all the model components. For components that are not conserved (i.e. subject to synthesis/degradation) including PYK2 and c-Met, their mRNA levels were adjusted by modifying the corresponding kinetic parameter (synthetic and degradation rates) values (*Vs5* and *kdeg6* for PYK2 mRNA, and *Vs13* and *kdeg14* for c-Met mRNA) based on implicit reverse functions such that these mRNA levels reflect the fold change of gene expression in each patient (see S20 Fig). The form of these functions: *Y = f(ks*,*kd)* (where *Y*, *ks* and *kd* denote the mRNA level, synthetic and degradation rate, respectively) was obtained numerically through model simulations (S20 Fig).

### Model-based computation of drug synergy indexes

Different drug synergy scores were used to numerically evaluate drug combination effect:

The Chou-Talalay’s Combination Index, (CI score) [32], Bliss Independence (BI score) (28) and Coefficient of Drug Interaction (CDI score) (29). Detailed description of these scores are given in S2 Text.

### Characterization of switch-like response

We adopted a Hill function to quantitatively characterize the switch-like features of the dose-response curves as follows (see S14 Fig):

$$Y=\frac{y_{\max}-y_{\min}}{1+{\left(\frac{I}{IC50}\right)}^{H}}+y_{\min},$$ (2)

where *Y* is a model readout (i.e., pERK or pSTAT3) and *y*_*max*_ and *y*_*min*_ are the maximum and minimum value of *Y*. *I* denotes the drug (Gefitinib and PF396). IC50 denotes the half-maximal concentration and *H* is the Hill coefficient.

### Data acquisition and processing for the patient stratification

A discovery patient cohort and an independent validation cohort were used for the patient stratification analyses. The former was obtained from The Broad Institute TCGA GDAC Firehose (gdac.broadinstitute.org), containing 108 patients with both patient-specific transcriptomic (RNA-seq) and proteomic (Reverse Phase Protein Array, RPPA) data for some proteins. As RPPA data was not available for all network nodes (state variables), RPPA data for selected nodes (EGFR, ERK and STAT3) were used in combination with gene expression data for the remaining nodes (PYK2, c-Met and CBL). The discovery cohort data (N = 108) was subclassified into different subtypes (Basal-Like1 (BL1, 34), Mesenchymal (M, 34), Luminal Androgen Receptor (LAR, 26), and unclassified (UNC, 1)) using the Vanderbilt subtyping system [40]. The independent cohort for validation was obtained from the European-Genome Phenome Archive (accession number EGAS00001001178) [60], consisting of 75 TNBC patient samples that were further subclassified into different subtypes (Basal-like 1 (BL1, 8), Basal-like 2 (BL2, 5), Immunomodulatory (IM, 16), mesenchymal stem-like (MSL, 6), Mesenchymal (M, 17), Luminal Androgen Receptor (LAR, 2), unspecified (UNS, 10) [40]. Several patient samples (PR11327a, PR11750a, PR4845a.RNA, PR4975a, PR5930a, PR5932a, PR5942a, PR6408a, PR6722a and PR9758a) were removed from the validation cohort since the Vanderbilt subtyping system [40] indicates these are potentially ER-positive. Consequently, a total of 65 TNBC patients were used for the validation cohort.

## Supporting information

S1 Text Model training and numerical simulations. (DOCX)

S2 Text Model-based computation of drug synergy index. (DOCX)

S3 Text Generation of patient-specific models. (DOCX)

S1 Fig Additional training and independent validation data. (a-b) These c-Met inhibition data was also used for model training. Model simulations (a) compare control condition, EGF stimulation and EGF + c-Met inhibition (by the inhibitor PHA) for different readouts: phospho-c-Met (pcMet), total c-Met (cMet), phospho-PYK2 (pPYK2) and total PYK2 expression. Corresponding experimental data (b) obtained from MDA-MB-468 cells are consistent with model predictions. These data was quantified from our previous study [16]. (c-d) Independent model validation: model simulations (c) comparing control condition and constitutive active STAT3 (STAT3CA) condition for phospho-STAT3 and total PYK2 expression. Corresponding experimental data (d) obtained from MDA-MB-468 cells are consistent with model predictions. These data was quantified from our previous study [16].
(TIF)

S2 Fig Additional evaluation of the best-fitted parameter set. An objective function metric (i.e. M, see the Methods section, main text for details) was used to evaluate the model accuracy in comparison with experimental data. Here, 1000 parameter sets were randomly generated within various ranges (2, 5 and 10 folds) of the fitted values and the model accuracy was evaluated. The results show that the best-fitted set consistently outperforms other randomized sets within its vicinity (also see the Methods section, main text for more details).
(TIF)

S3 Fig Illustration of a drug dose-response curve. ICX (X = 0–100) is defined as the drug concentration at which the corresponding model output (e.g. signaling readouts pERK/pSTAT3) is inhibited by X%, as compared the untreated condition (no drug). Illustration of IC25, IC50 and IC75 were depicted.
(TIF)

S4 Fig Model simulations of the effects of single-drug vs. combined-drug treatments for six drug combinations considered in this study, on pERK and pSTAT3 (normalised by total levels) as the signaling readouts. D1: first drug, D2: second drug in the same order as in the x-axis’s label. Concentration at IC_35_ values was used for each drug.
(TIF)

S5 Fig **Predicted level of synergy** (synergy index, log2 scale) for each drug combination using the Chou-Talalay based CI scores (see main text and Methods for details) (a-b) Gefitinib associated parameter (Ki1) value was perturbed 50%. (c-d) PF396 associated parameter (Ki3b) was perturbed 50%.
(TIF)

S6 Fig Predicted levels of synergy computed for each drug combinations using both the Bliss Independent (BI) and Combination Drug Index (CDI) methods, in log2 scale (see main text and Methods for details). Concentrations at IC_25_ values were used for BI and CDI, respectively.
(TIF)

S7 Fig Predicted levels of synergy computed for each drug combinations using both the Bliss Independent (BI) and Combination Drug Index (CDI) methods, in log2 scale (see main text and Methods for details). Concentrations at IC_50_ values were used for BI and CDI.
(TIF)

S8 Fig Drug combination matrices. Inhibition effects on cell growth are depicted as percentage relative to non-treated cells. Colour gradient code: from untreated (green) to strongly inhibited (red).
(TIF)

S9 Fig Switch-like responses of pERK and pSTAT3. **(a-b)** 3-dimensional (3D) simulations of the (normalised) steady-state pERK/pSTAT3 levels to simultaneous increases in EGF and HGF stimulation, displaying an switch-like pattern. **(c-d)** 2D dose-response simulations of steady-state pERK/pSTAT3 to Gefitinib and PF396, respectively. H denotes the fitted Hill coefficient of the dose response curve.
(TIF)

S10 Fig Data quantification and dose-dependent effect of PF396. **(a-b)** Quantification of Western blot data in Fig 3C, main text. **(c-d)** Simulated time profile of pERK and pSTAT3. Model simulations show dose-dependent effect of PF396-mediated PYK2 inhibition on the response of STAT3 and ERK activities. **(e)** Western blot data measuring signalling responses to increasing doses of PF396. **(f-g)** Quantification of the data in (e), control was the same as in Fig 3C, main text.
(TIF)

S11 Fig EGFR phosphorylation in response to increasing Gefitinib dosage, showing 1μM is the minimum concentration that efficiently inhibits EGFR phosphorylation. To find the lowest usable concentration of Gefitinib to inhibit EGFR phosphorylation, cells were treated with gradually increasing concentrations of the inhibitor for 24 hrs. Phosphorylated and total protein levels were assayed by Western Blot.
(TIF)

S12 Fig Three-dimensional (3D) dose-response profiles of pERK/pSTAT3 to simultaneous increase in concentrations of six considered drug combinations. (TIF)

S13 Fig Drug-mediated switch-like responses. (a,b) Simulated dose-response of pERK/pSTAT3 to increasing PF396 in the absence or presence of Gefitinib pre-treatment, showing Gefitinib increased cancer cell sensitivity to PF396. (c) Quantified IC50 values of PF396 in the absence or presence of Gefitinib pre-treatment. (d,e) Cell viability in response to PF-396 in the absence or presence of Gefitinib, measured in MDA-MB-468 and BT-20 cells [12]. Pre-treatment with Gefitinib significantly increased drug sensitivity to PF396 as predicted. (f) Quantified IC50 values of PF396 in the absence or presence of Gefitinib pre-treatment. (g,h) Simulated dose-response of pERK/pSTAT3 to increasing Gefitinib in the absence or presence of PF396 treatment, which sensitised cancer cells to Gefitinib. (i) Quantified IC50 values of Gefitinib in the absence or presence of PF396 treatment. (j,k) Cell viability in response to Gefitinib in normal or PYK2-deficient cells measured in MDA-MB-468 and BT-20 cells by colony formation assay [12]. Combined treatment with PF396 significantly increased drug sensitivity to Gefitinib. (l) Quantified IC50 values of Gefitinib in the absence or presence of PF396 treatment.
(TIF)

S14 Fig Characterization of dose-response curve by fitting to Hill function. The fitted Hill coefficient (*H*) signifies the switch-ness (steepness) of the response curve: a higher *H* value shows that the response curve is more switch-like (steep). y_max_ and y_min_ are the maximum and minimum value of the output response. On the other hand, IC50 denotes the half-maximal concentration and signifies the sensitivity of the output to the inhibitor (*I*).
(TIF)

S15 Fig Correlation between protein and RNA expression of signalling nodes. Correlation analysis of >1000 BC patients from TCGA [39] revealed relatively strong correlations between protein and RNA expression levels of signalling molecules that have both types of data available, suggesting RNA levels can serve as a good proxy for protein expression.
(TIF)

S16 Fig Predicted drug synergy for Gefitinib+PF396 co-treatment in patients as grouped by Vanderbilt-based TNBC subtyping system. The discovery **(a)** and validation cohort **(b)** datasets both show no significant differences in the predicted synergy index (assessed by BI scores) between the patients classified using the Vanderbilt-based subtyping system. The computed synergy indexes for Gefitinib + PF396 co-treatment of statistical significance were calculated based on analysis of variance (ANOVA).
(TIF)

S17 Fig Patient stratification analysis using an independent validation patient cohort. The validation TNBC cohort data were first clustered into Vanderbilt-based subtypes (upper panel). Synergy indices (BI scores) of EGFR and PYK2 co-inhibition predicted for each patient using the patient-specific network model (lower panels) are depicted. Only patients displaying sensitivity to Gefitinib and PF396 alone are included for synergy analysis for the combined treatment (i.e. 42 and 50 patients for pERK and pSTAT3 as readouts, respectively). IC50 concentrations were used for each drug. **(a-b)** Stratification analysis with pERK as readout. Patient samples sorted by the predicted synergistic effect. Q1 and Q3 denote the ‘synergistic’ and ‘antagonistic’ patient subgroups, respectively. Q1 and Q3 contain patients having synergy index values in the lower quartile and in the upper quartile, respectively. **(c-d)** Stratification analysis with pSTAT3 as readout as in panels (a-b). **(e-f)** Differential gene expression analysis between the Q1 and Q3 subgroups, showing upregulation of PYK2 is statistically significant in Q1 subgroup for both signalling readouts. *P<0.05; **P<0.01.
(TIF)

S18 Fig Heatmap showing a ‘reference’ patient sample molecularly most similar to MDA-MB-468 cell line. Using gene expression data, hierarchical clustering method identified that the expression profile of the sample ID TCGA-E2-A1LG-01 within the discovery cohort is most similar to that of the MDA-MB-468 cell line. This patient then serves as a reference for normalising data as model inputs to generate patient-specific models.
(TIF)

S19 Fig Patient stratification analysis using the discovery cohort with alternative normalisation method. The discovery TNBC cohort data were normalized by the patient sample whose molecular profile is most similar to that of the MDA-MB-468 cell line using hierarchical clustering method (S18 Fig). The normalized patient samples were first clustered into four Vanderbilt based subtypes. Synergy indices (BI scores) of EGFR and PYK2 co-inhibition predicted for each patient using the patient-specific network model (lower panels) are depicted. Only patients displaying sensitivity to Gefitinib and PF396 alone are included for synergy analysis in combined treatment (i.e. 48 and 57 patients for pERK and pSTAT3 as readouts, respectively). IC50 concentrations were used for each drug. **(a-b)** Stratification analysis with pERK as readout. Patient samples sorted by the predicted synergistic effect. Q1 and Q3 denote the ‘synergistic’ and ‘antagonistic’ patient subgroups, respectively. Q1 and Q3 contain patients having synergy index values in the lower quartile and in the upper quartile, respectively. **(c-d)** Stratification analysis with pSTAT3 as readout as in panels (a-b). **(e-f)** Differential gene expression analysis between the Q1 and Q3 subgroups, showing upregulation of PYK2 is statistically significant in Q1 subgroup for both signalling readouts. *P<0.05; **P<0.01.
(TIF)

S20 Fig **Implicit reverse functions** used for adjustment of kinetic parameter values of PYK2 **(a)** and c-Met mRNA **(b)**. In order to generate patient specific network model, we adjusted mRNA levels of PYK2 and c-Met by modifying kinetic parameter values (*Vs5* and *kdeg6* for PYK2 mRNA, and *Vs13* and *kdeg14* for c-Met mRNA) based on implicit reverse functions. For example, if the tumour sample showed a 30 fold increase in PYK2 gene expression, then we modified kinetic parameter values of *kdeg6* = 280 and *Vs5* = 52 based on the implicit reverse function.
(TIF)

S1 Table Reactions and reaction rates of the EGFR-c-MET-PYK2 network model. (DOCX)

S2 Table Ordinary differential equations of the EGFR-c-MET-PYK2 model. The reaction rates are given in S1 Table.
(DOCX)

S3 Table Best-fitted parameter values used for simulations. (DOCX)

S4 Table Parameters perturbed for feedback functional analysis shown in Fig 3H–3O, Fig 5D–5O. (DOCX)

S5 Table Algebraic constraints used for parameter estimation. As described in the Methods section, main text, the following qualitative constraints were incorporated into the overall objective function M for parameter estimation.
(DOCX)

S6 Table Reported mutations related to the EGFR-PYK2-cMET network within the discovery patient cohort. Out of 108 TNBC patients with available mutation information, we identified 10 patients who have reported mutations in any of the network nodes. Specifically, four patients contain EGFR mutations, two contain PYK2 mutations, another two contain STAT3 mutations, and Met and Cbl mutation were found in one single patient. The most common mutation types are SNP. However, searching for these mutations in the COSMIC mutation catalogue (http://cancer.sanger.ac.uk/cosmic), the most comprehensive mutation database currently available, we found mostly no functional effects (i.e. GOF or LOF) reported for these genetic changes, except that STAT3 H410R (indicated by an asterisk) is associated with induction of STAT3 phosphorylation and SOCS3, CCL2, JUNB and BCL3 upregulation in large granular lymphocyte (LGL) leukemia [61]. Therefore, within the EGFR-PYK2-cMET network, the majority of the considered TNBC patients displayed no functionally significant mutation events. Rather, the heterogeneity of the patient landscape primarily comes from alteration in expression levels.
(DOCX)

S1 File The SBML model file. (XML)
