# Supplementary material for: Systems modelling of the EGFR-PYK2-c-Met interaction network predicts and prioritizes synergistic drug combinations for triple-negative breast cancer
Source: PLoS Comput Biol. 2018 Jun 19;14(6):e1006192. doi: 10.1371/journal.pcbi.1006192 (PMC6007894; doi:10.1371/journal.pcbi.1006192)
Supplement: S1 Text — (DOCX) [file pcbi.1006192.s001.docx]

**S1. Model training and numerical simulations**

Model training (or calibration) is a process that aims to fit a computational model to known and quantified experiment data whereby unmeasured model parameters (including kinetic and state variables) are estimated so that model simulations can recapitulate the data. Essentially, parameter estimation is an optimisation problem in which a carefully formulated objective function representing the discrepancy between model simulations and experimental data is minimised.

*Objective function:*

In this study, the following metric function (also called ‘cost function’) was used to quantify the discrepancy between simulated values and corresponding experimental measurements [1-3] :

$$J(p)=\sum_{j=1}^{m} \sum_{i=1}^{n} \left(\frac{y_{j,i}^{D}-y_{j}(t_{i},p)}{\sigma_{j,i}}\right)^{2}$$

where *m* is the number of the given experimental data sets used for fitting and *n* is the number of time points within each experimental data set. $y_{j}(t_{i},p)$ represents the numerical solution for the model state variable $y_{j}$evaluated at time $t_{i}$ and parameter set $p$; while$y_{j,i}^{D}$ is the corresponding data point at $t_{i}$ with the associated error variance$\sigma_{j,i}$. For normally distributed measurement errors, this metric function corresponds to a Maximum Likelihood estimation [1].

It is important to note that in many cases including this study [1-3], the data used for model fitting are often obtained by semi-quantitative techniques, for instance Western blotting or reverse transcription polymerase chain reaction (RT-PCR), and represent relative levels of signal intensity. As both the relative data (e.g. phosphorylated level/total level) and the corresponding simulated values are used in the metric function, minimising the metric function alone could produce a good fit between simulations and data, the fitted parameter sets however may yield biologically unreasonable behaviours. For examples, the level of phosphorylated protein in the model may account for just a very small fraction (<0.01%) of the total protein. Thus, to avoid such potential scenarios, we further incorporated a number of algebraic constraints (*R_i_, i=1…3,* supplementary Table S5) on the dynamic variables of the model, e.g. to ensure that phosphorylated protein abundances are within a reasonable ranges in comparison to their total abundances.

The new objective function, M, was then formed by combining the metric function *J* and the algebraic functions *R*s (see Table S5), which was then used for the model calibration:

$$M(p)=J(p)+\sum_{i=1}^{3} R_{i}(p)$$

*Genetic Algorithm for optimizing the objective function*

In this study, a Genetic Algorithm (GA) was used for parameter estimation [2,3] since GA is particularly suited for optimization problems [2,4,5]. Genetic algorithms is a class of optimisation algorithm inspired by the process of natural selection central to biological evolution [2,3,5,6]. As such, GA repeatedly searches for and updates a population of candidate solutions (called individuals or phenotypes). Usually, the initial population is randomly generated, covering the entire range of possible solutions (called ‘search space’). The population size depends on the nature of the problem, but typically contains several hundreds to thousands of possible solutions. At each step, GA selects individuals from the existing population to breed a new generation. During successive generations, the population "evolves" toward an optimal solution. Sometimes, GA may have a tendency to converge toward local minima. In this case, we can repeat the GA process by increasing the population size and/or changing the mutation and crossover rates. GA is equipped with three main biologically-inspired rules to generate the next generation of parameter values from the current population: selection, crossover and mutation.

For this work, we implemented GA using the Global Optimization Toolbox and the function *ga* in MATLAB ® (The MathWorks. Inc. 2016b). Selection rules select the individual solutions with the best fitness values (called ‘elite solution’) from the current population. The elite count was set to 5% of the population size. Crossover rules combine two parents to generate offspring for the next generation. The crossover faction was set at 0.8. Note that for the setting of the elite rate and the crossover faction we used conventional values most commonly used in GA and also in our previous studies [2,3]. Mutation rules apply random changes to individual parents to generate the population of the next generation. For the mutation rule, we generated a random number from a Gaussian distribution with mean 0 and standard deviation σ_k_, which was applied to the individuals of the current generation. The standard deviation function (σ_k_) is given by the recursive formula as follows:

$\sigma_{k}=\sigma_{k-1}\left(1-\frac{k}{G}\right)$,

where *k* is the *k*^th^ generation, *G* is the number of generation, and $\sigma_{0}=1$.

*Numerical implementation*

The model training procedures and all numerical simulations in this study were implemented using MATLAB. The computationally intensive model training was performed on a dedicated High Performance Computing (HPC) cluster available to our group at Monash University (<http://www.monash.edu>). This HPC facility consists of two Haswell CPU sockets with a total of 16 physical cores (or 32 hyperthreaded cores) at 3.20 GHz and 300 TB usable storage. Similarly, the patient-specific modelling and simulations, as well as calculation of the drug synergy indexes for each patient were also computationally intensive and were thus performed using the same HPC cluster. Less intensive, e.g. time-course simulations, were performed using Matlab on a desktop PC (3.4 GHz quad core Intel i7-6700).

The Ordinary differential equations (ODEs) were integrated and solved using the function *ode15s* in Matlab, which is a variable-step and variable-order solver, based on the numerical differentiation formulas (NDFs) and is specially designed for stiff systems.

*Identification of the best fitted parameter set*

To derive the best fitted parameter set which was subsequently used for simulations in this study, we carried out the repeated GA runs with 5,000 of the population size and 100 of the generation number. In this computation, we also changed the mutation and crossover rates and even the population size to escape from trapping in local minima. After multiple repetitions of the GA process where the best fitted set obtained from a previous repeat was used as the starting point of the next repeat, we arrived at the final best fitted set as the objective function was not further reduced, and the fitted parameter values no longer change. This gave us confidence that the used parameter set represents the optimal set that minimise the simulations/data discrepancies. To further show that this is the case, we have now performed additional analysis comparing the performance of the best fitted set against other randomised parameter sets positioned in the vicinity of the best fitted set in the multi-dimensional parameter space. Specifically, we generated a total of 1000 parameter sets by perturbing each parameter randomly within a 2-fold range around their corresponding best fitted values. The objective function M was then recomputed for all 1000 sets and compared with that of the current best-fitted one. Figure S2 shows that our current set displays the lowest objective function value, suggesting it indeed represent the optimal set. Moreover, when we enlarged the ranges for drawing the random parameter values (e.g. 2-fold, 5-fold and 10-fold, displayed in Fig. S2), the current best-fitted set consistently performed better than the randomised sets.

**SUPPLEMENTARY REFERENCE**

1. Raue A, Kreutz C, Maiwald T, Bachmann J, Schilling M, et al. (2009) Structural and practical identifiability analysis of partially observed dynamical models by exploiting the profile likelihood. Bioinformatics 25: 1923-1929.

2. Shin SY, Kim T, Lee HS, Kang JH, Lee JY, et al. (2014) The switching role of β-adrenergic receptor signalling in cell survival or death decision of cardiomyocytes. Nat Commun 5: 5777.

3. Shin D, Kim IS, Lee JM, Shin SY, Lee JH, et al. (2014) The hidden switches underlying RORalpha-mediated circuits that critically regulate uncontrolled cell proliferation. J Mol Cell Biol 6: 338-348.

4. Reali F, Priami C, Marchetti L (2017) Optimization Algorithms for Computational Systems Biology. Frontiers in Applied Mathematics and Statistics 3.

5. Man KF, Tang KS, Kwong S (1996) Genetic algorithms: concepts and applications [in engineering design]. IEEE Transactions on Industrial Electronics 43: 519-534.

6. Srinivas M, Patnaik LM (1994) Genetic algorithms: a survey. Computer 27: 17-26.
