# Supplementary material for: Systems modelling of the EGFR-PYK2-c-Met interaction network predicts and prioritizes synergistic drug combinations for triple-negative breast cancer
Source: PLoS Comput Biol. 2018 Jun 19;14(6):e1006192. doi: 10.1371/journal.pcbi.1006192 (PMC6007894; doi:10.1371/journal.pcbi.1006192)
Supplement: S2 Text — (DOCX) [file pcbi.1006192.s002.docx]

**S2. Model-based computation of drug synergy index**

Different drug synergy scores were used to numerically evaluate drug combination effect: the Chou-Talalay’s Combination Index, (CI score) [1], Bliss Independence (BI score) (28) and Coefficient of Drug Interaction (CDI score) (29). These are explained below.

The coefficient of drug interaction (CDI) is a simple model to the synergistically inhibitory effect of the drug combination [2,3]. CDI is calculated as follows: CDI=*E_12_*/(*E_1_*×*E_2_*), where *E_12_* is a normalized biological response (e.g., cell survival) at combination treatment of Drug A and Drug B by its control group, and *E_1_* and *E_2_* are the response measured at single drug treatment, respectively. CDI <1, = 1 or >1 indicates that the drugs are synergistic, additive or antagonistic, respectively. For instance, if cell survival is inhibited 50% by a combined drug treatment, and 30% and 20% inhibited by single drug treatment, respectively, then we have CDI=(1-0.5)/((1-0.3)×(1-0.2))=0.89, implying that the combined treatment has a synergistic effect.

Bliss Independence (BI) is another statistical model to assess the combination efficacy of two drugs based on the assumption that the individual drugs do not directly interfere with each other and drugs contribute to a common result [4,5]. The combined activity ($E_{12}^{Pred}$) at concentration C_1_ and C_2_ can be predicted using the complete activity of probability theory [5] as

$$E_{12}^{Pred}=f_{1}+f_{2}-f_{1}{\cdot f}_{2}.$$

BI is defined as follows: BI=$E_{12}^{Pred}$/$E_{12}^{Obs}$, where $E_{12}^{Obs}$denotes the observed combined effect at concentration C_1_ and C_2_. For instance, if cell survival is inhibited 50% by a combined drug treatment, and 30% and 20% inhibited by single drug treatment, respectively, then we have $E_{12}^{Pred}=0.3+0.2-0.3\times0.2=0.44$ and $E_{12}^{Obs}=0.5$, thus BI=0.44/0.5=09. BI <1, = 1 or >1 indicates that the drugs are synergistic, additive or antagonistic, respectively.

The combination index (CI) is the preferred additive reference model devised by Loewe and generalized by Chou and Talalay for analysing combination effects based on the principle of mass action [4,6]. The central assertion of this model is that a compound must be addictive when combined with itself [4]. *CI_x_* for quantification of synergism or quantification for two drugs is calculated as follows:

${CI}_{x}=\frac{\left( D \right)_{1}}{{(D_{x})}_{1}}+\frac{\left( D \right)_{2}}{{(D_{x})}_{2}} =\frac{\left( D \right)_{1}}{{(D_{m})}_{1}\left[ \frac{f_{a}}{\left( 1-f_{a} \right)} \right]^{1/{m_{1}}}}$ +$\frac{\left( D \right)_{2}}{{(D_{m})}_{2}\left[ \frac{f_{a}}{\left( 1-f_{a} \right)} \right]^{1/{m_{2}}}},$

where (*D_x_*)_1_ is the concentration of *Drug 1* alone that inhibits a system *x*%, and (*D_x_*)_2_ is the concentration of *Drug 2* alone that inhibits a system *x*%. (D)_1_ and (D)_2_ are the concentrations that inhibits x% in combination. The (*Dm*)*_i_* and *m_i_*, *i*=1,2 values can be determined by fitting the median-effect equation (that describes dose-effect relation) of the individual compound to measured experimental data [6].

**SUPPLEMENTARY REFERENCE**

1. Chou TC (2010) Drug combination studies and their synergy quantification using the Chou-Talalay method. Cancer Res 70: 440-446.

2. Li X, Lin Z, Zhang B, Guo L, Liu S, et al. (2016) β-elemene sensitizes hepatocellular carcinoma cells to oxaliplatin by preventing oxaliplatin-induced degradation of copper transporter 1. Scientific Reports 6: 21010.

3. Liu F, Shang Y, Chen S-z (2014) Chloroquine potentiates the anti-cancer effect of lidamycin on non-small cell lung cancer cells in vitro. Acta Pharmacol Sin 35: 645-652.

4. Keith CT, Borisy AA, Stockwell BR (2005) Multicomponent therapeutics for networked systems. Nature reviews Drug discovery 4: 71-78.

5. Zhao W, Sachsenmeier K, Zhang L, Sult E, Hollingsworth RE, et al. (2014) A New Bliss Independence Model to Analyze Drug Combination Data. Journal of Biomolecular Screening 19: 817-821.

6. Chou TC (2006) Theoretical basis, experimental design, and computerized simulation of synergism and antagonism in drug combination studies. Pharmacological reviews 58: 621-681.
