# Supplementary material for: Systems modelling of the EGFR-PYK2-c-Met interaction network predicts and prioritizes synergistic drug combinations for triple-negative breast cancer
Source: PLoS Comput Biol. 2018 Jun 19;14(6):e1006192. doi: 10.1371/journal.pcbi.1006192 (PMC6007894; doi:10.1371/journal.pcbi.1006192)
Supplement: S3 Text — (DOCX) [file pcbi.1006192.s003.docx]

**S3. Generation of patient-specific models.**

*Personalizing patient models by incorporating expression data*

The fact that the expression of all the species included in our model were detected across the patient samples suggest that the network nodes are shared between the patients. However, the network specifications (wirings) in terms of the strength of the links (i.e. connectivities) between the nodes were distinctly different between the patients due to the patient-specific values of the model state variables that were determined by expression data and incorporated in our models. For instances, patients with upregulated basal STAT3 would confer stronger feedback regulation emanating from STAT3 to PYK2 or to c-Met, whereas patients with low STAT3 levels would display weak regulation by these feedbacks. Such variations in the network wirings potentially lead to heterogeneous responses to the same therapy between patients. A clinically relevant example is that of the negative feedback loop from ERK to EGFR in cancers harbouring oncogenic BRAF(V600E) mutation [1]. While BRAF-mt colon/thyroid tumours displayed resistance to these drugs, BRAF-mt melanoma exhibited dramatic responses instead. This difference was actually due to the differential EGFR expressions between these tumours: EGFR are high in colon/thyroid cancers therefore the ERK-to-EGFR feedback is functional, and so inhibiting BRAF suppressed ERK, leading to activated EGFR (feedback relief) which stimulates compensatory oncogenic signals; whereas melanomas have very low EGFR expression in comparison, rendering the ERK-to-EGFR feedback non-functional and thus sensitive to BRAF inhibition [1,2]. This example demonstrates that differential expression profiles between patients significantly determine variable drug responses. Thus incorporating patient-specific expression profiles into our models allowed us to capture a major source of patient-to-patient variations. We however acknowledge that there are other factors that may contribute to variations in patient drug response such as the specific tumour microenvironment within each patient, however capturing these factors is beyond the scope of the current work.

*Combining transcriptomic and proteomic expression data for model generation*

We combined both RPPA (reverse phase protein array) based protein expression data and RNA-seq based gene expression data as model inputs to generate the patient-specific models. Compared to the wide availability of patient RNA-seq data, proteomic data obtained from patients are comparatively much rarer due to technical challenges. Thus, a major reason why we resorted to patient RNA expression data for the generation of patient-specific models and subsequent patient stratification was because of the lack of patient data at the proteomic level. Even if these data are available (typically by reverse phase protein array, RPPA), they have low coverage meaning only a handful of our network nodes are included in the antibody panel, as opposed to the genome-wide coverage of RNA-seq data, making RPPA data less suitable for keeping model consistency. In further support of our argument, correlation analysis of >1000 BC patients from TCGA [3] showed good correlations between protein and RNA expression levels of representative signalling molecules which have both types of data available (see Fig. S15), suggesting RNA level can serve as a proxy for protein expression. Moreover, previously published work [4] provide further evidence that model simulations using RNA expression data can yield reliable predictions which were validated experimentally. Thus while being constrained by the lack of patient proteomic data, the utilisation of RNA data in combination with protein expression data for modelling and simulations in a patient setting was a pragmatic approach.

**SUPPLEMENTARY REFERENCE**

1. Prahallad A, Sun C, Huang S, Di Nicolantonio F, Salazar R, et al. (2012) Unresponsiveness of colon cancer to BRAF(V600E) inhibition through feedback activation of EGFR. Nature 483: 100-103.

2. Nguyen LK, Kholodenko BN (2015) Feedback regulation in cell signalling: Lessons for cancer therapeutics. Seminars in cell & developmental biology 10.1016/j.semcdb.2015.09.024: 85-94.

3. Ciriello G, Gatza ML, Beck AH, Wilkerson MD, Rhie SK, et al. (2015) Comprehensive Molecular Portraits of Invasive Lobular Breast Cancer. Cell 163: 506-519.

4. Fey D, Halasz M, Dreidax D, Kennedy SP, Hastings JF, et al. (2015) Signaling pathway models as biomarkers: Patient-specific simulations of JNK activity predict the survival of neuroblastoma patients. Science signaling 8: ra130.
