# Supplementary figures and images for: Systems modelling of the EGFR-PYK2-c-Met interaction network predicts and prioritizes synergistic drug combinations for triple-negative breast cancer
Source: PLoS Comput Biol. 2018 Jun 19;14(6):e1006192. doi: 10.1371/journal.pcbi.1006192 (PMC6007894; doi:10.1371/journal.pcbi.1006192)

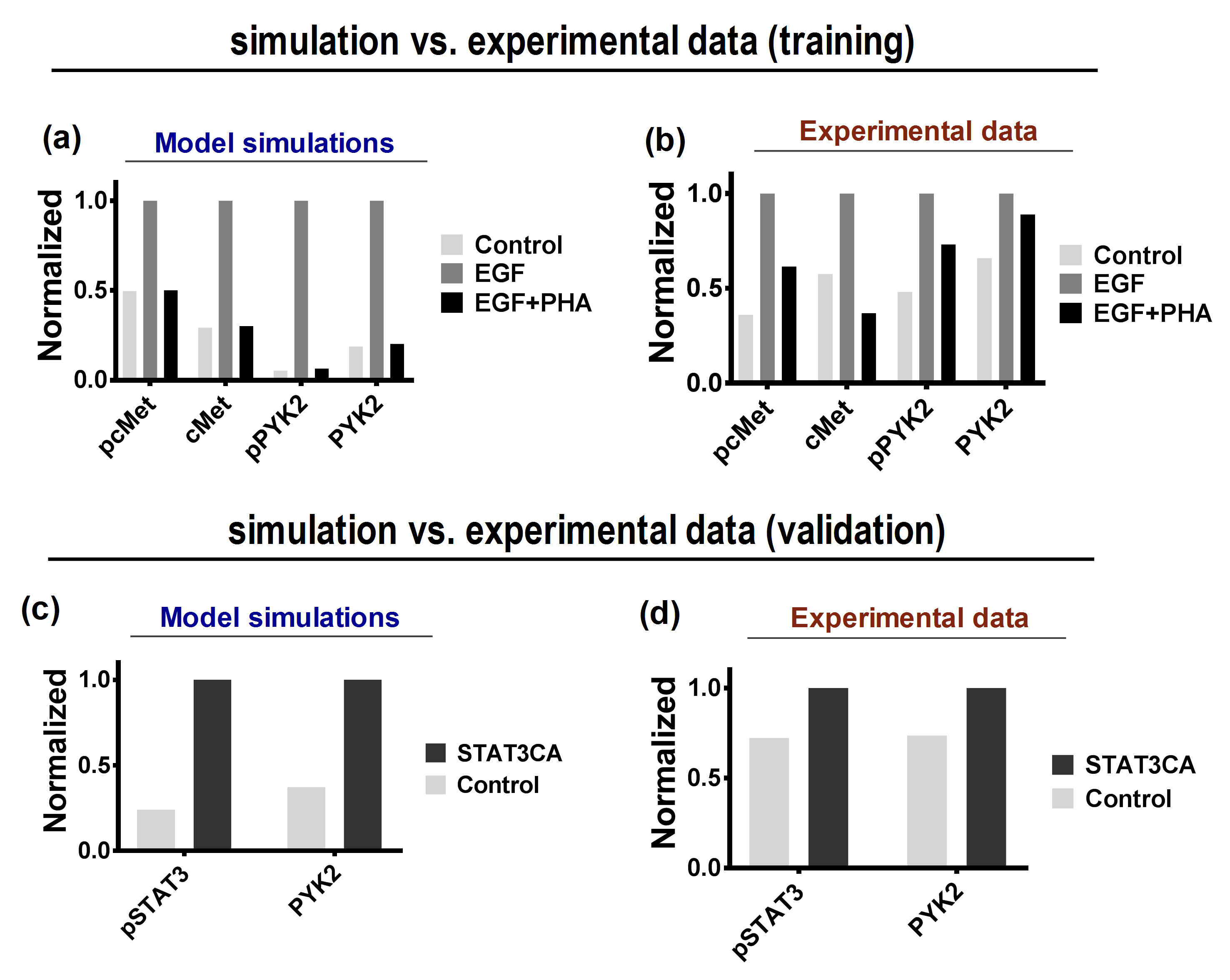

Supplement: S1 Fig — (a-b) These c-Met inhibition data was also used for model training. Model simulations (a) compare control condition, EGF stimulation and EGF + c-Met inhibition (by the inhibitor PHA) for different readouts: phospho-c-Met (pcMet), total c-Met (cMet), phospho-PYK2 (pPYK2) and total PYK2 expression. Corresponding experimental data (b) obtained from MDA-MB-468 cells are consistent with model predictions. These data was quantified from our previous study [16]. (c-d) Independent model validation: model simulations (c) comparing control condition and constitutive active STAT3 (STAT3CA) condition for phospho-STAT3 and total PYK2 expression. Corresponding experimental data (d) obtained from MDA-MB-468 cells are consistent with model predictions. These data was quantified from our previous study [16]. (TIF) [file pcbi.1006192.s004.tif]

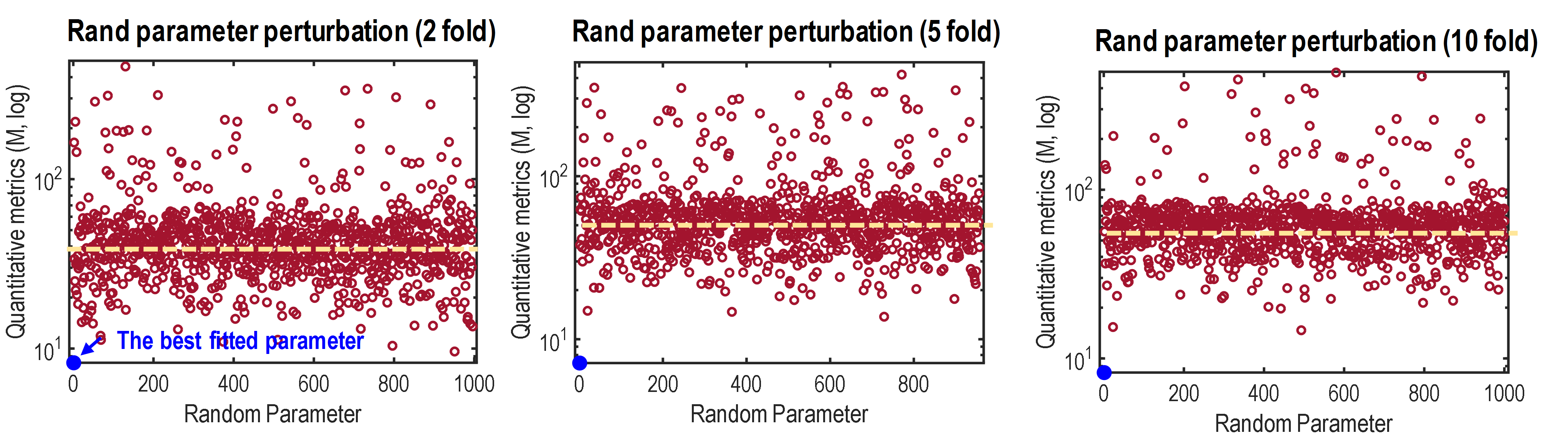

Supplement: S2 Fig — An objective function metric (i.e. M, see the Methods section, main text for details) was used to evaluate the model accuracy in comparison with experimental data. Here, 1000 parameter sets were randomly generated within various ranges (2, 5 and 10 folds) of the fitted values and the model accuracy was evaluated. The results show that the best-fitted set consistently outperforms other randomized sets within its vicinity (also see the Methods section, main text for more details). (TIF) [file pcbi.1006192.s005.tif]

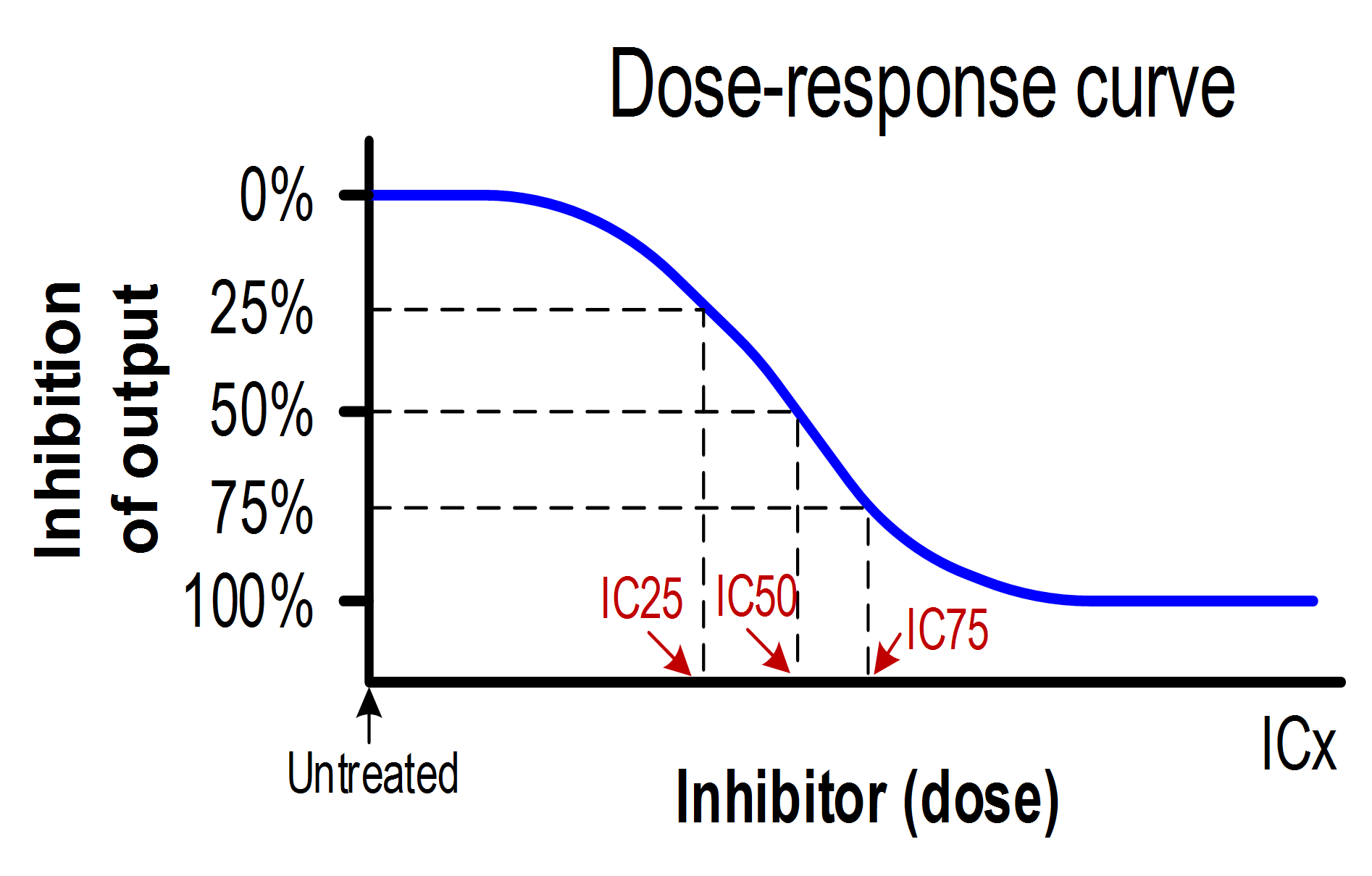

Supplement: S3 Fig — ICX (X = 0–100) is defined as the drug concentration at which the corresponding model output (e.g. signaling readouts pERK/pSTAT3) is inhibited by X%, as compared the untreated condition (no drug). Illustration of IC25, IC50 and IC75 were depicted. (TIF) [file pcbi.1006192.s006.tif]

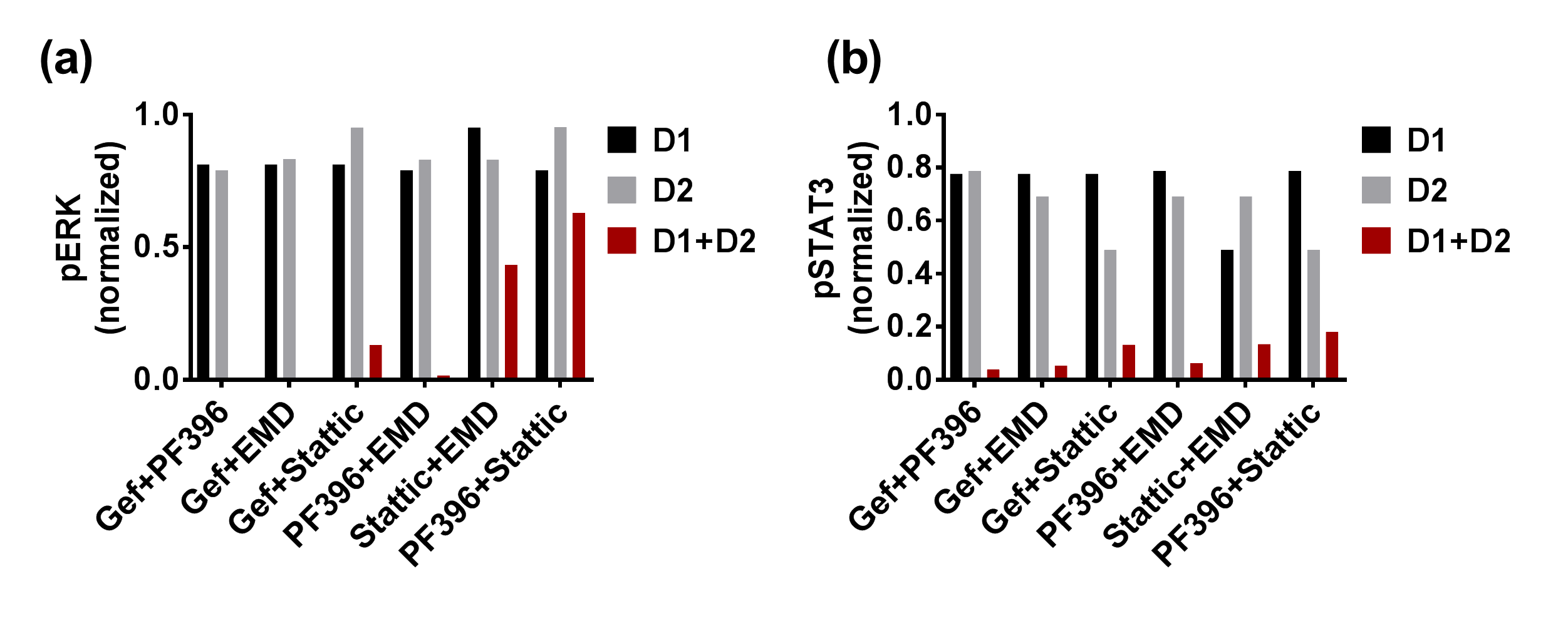

Supplement: S4 Fig — D1: first drug, D2: second drug in the same order as in the x-axis’s label. Concentration at IC35 values was used for each drug. (TIF) [file pcbi.1006192.s007.tif]

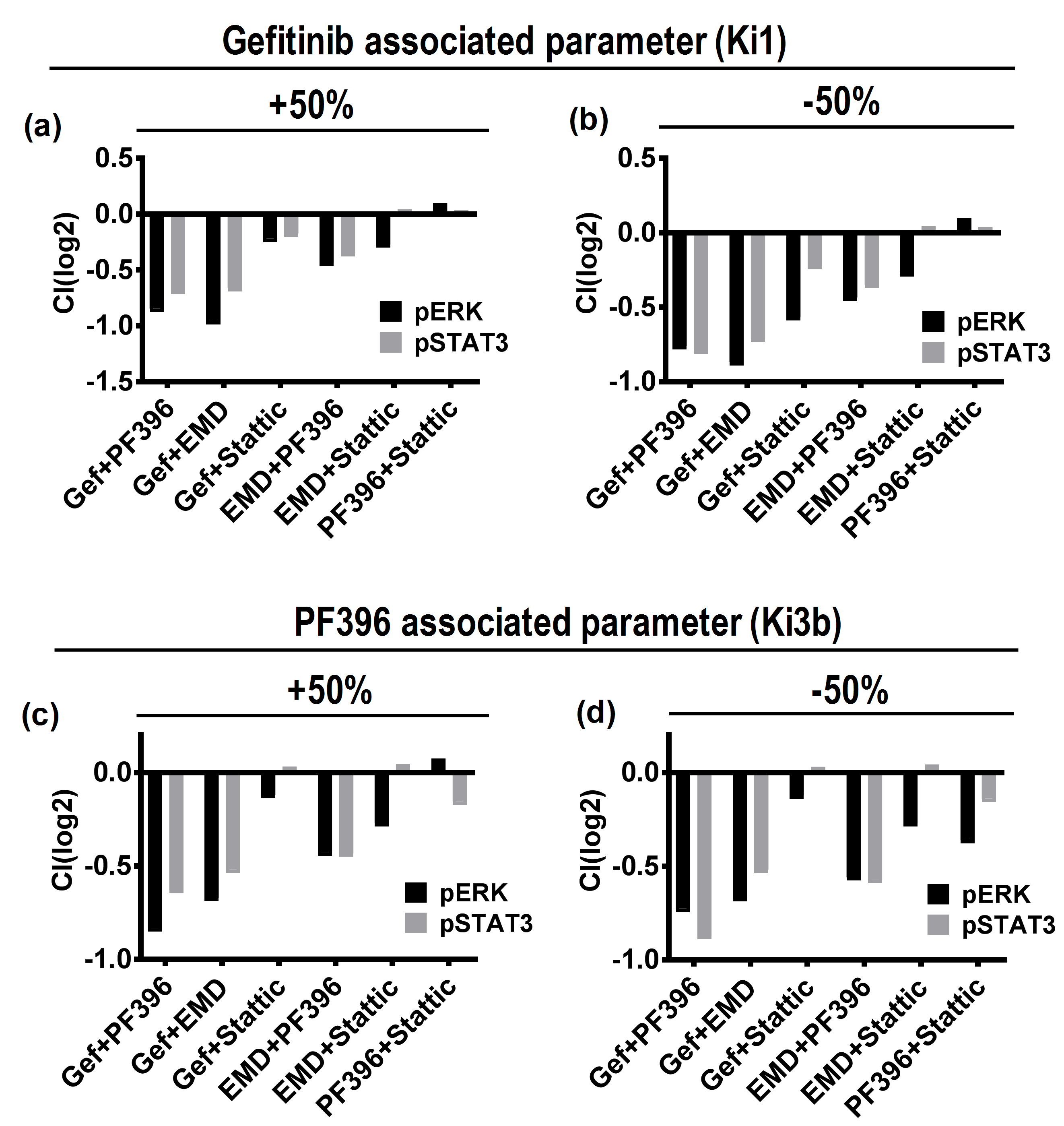

Supplement: S5 Fig — Predicted level of synergy (synergy index, log2 scale) for each drug combination using the Chou-Talalay based CI scores (see main text and Methods for details) (a-b) Gefitinib associated parameter (Ki1) value was perturbed 50%. (c-d) PF396 associated parameter (Ki3b) was perturbed 50%. (TIF) [file pcbi.1006192.s008.tif]

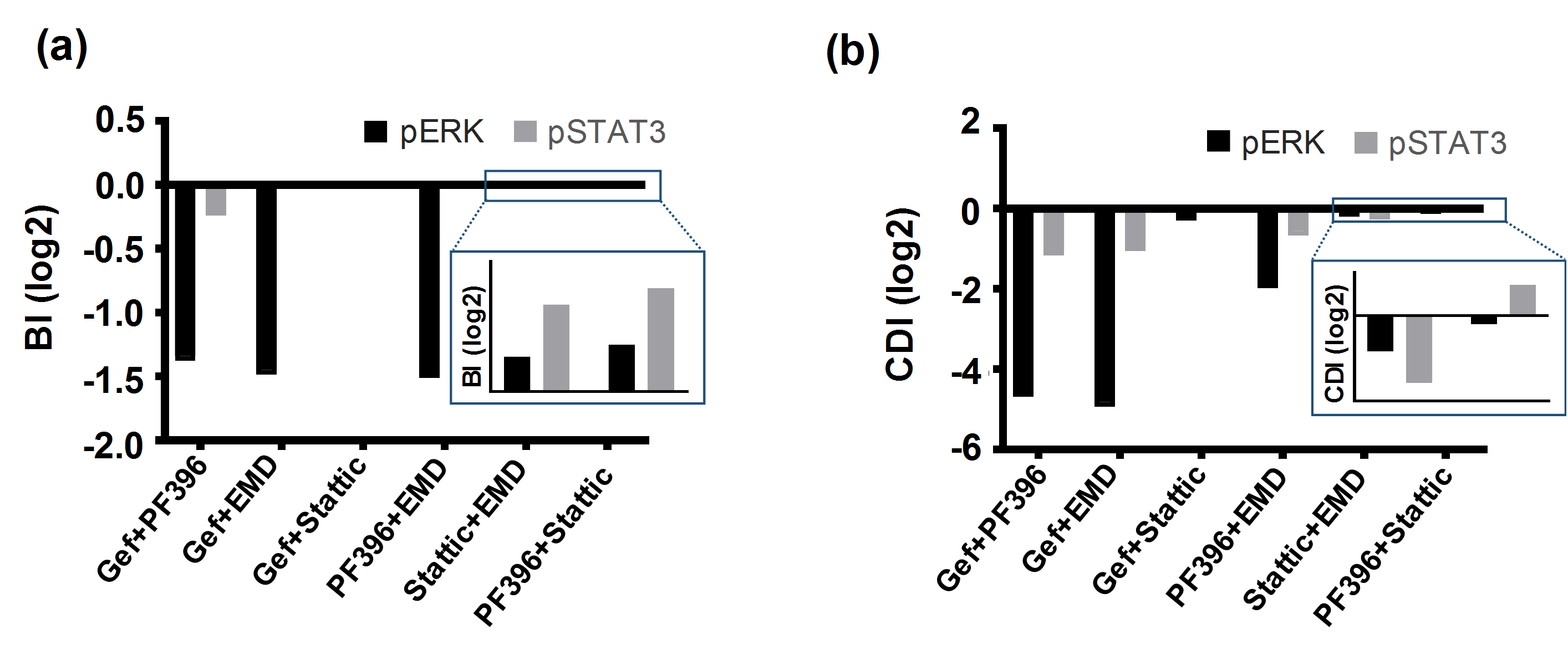

Supplement: S6 Fig — Concentrations at IC25 values were used for BI and CDI, respectively. (TIF) [file pcbi.1006192.s009.tif]

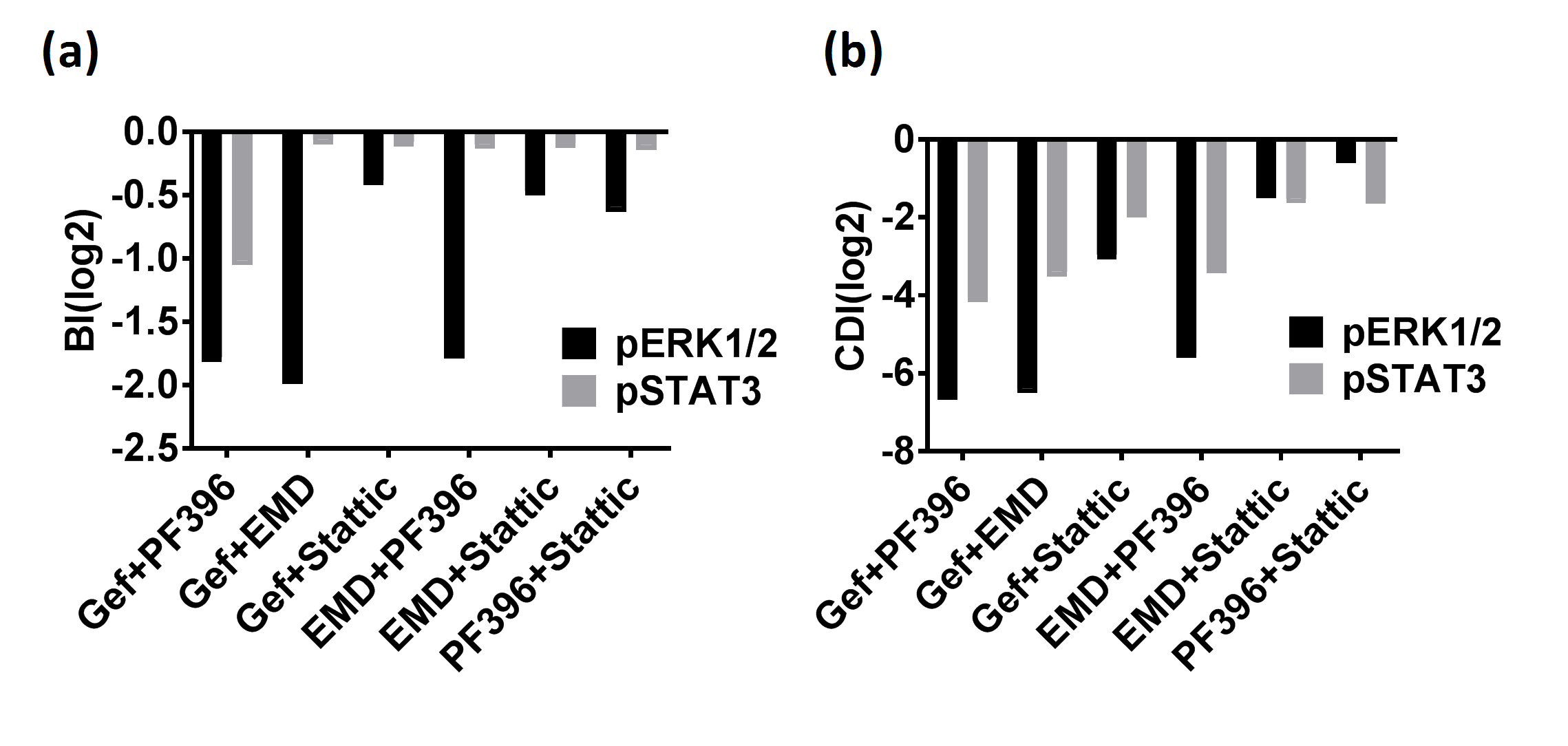

Supplement: S7 Fig — Concentrations at IC50 values were used for BI and CDI. (TIF) [file pcbi.1006192.s010.tif]

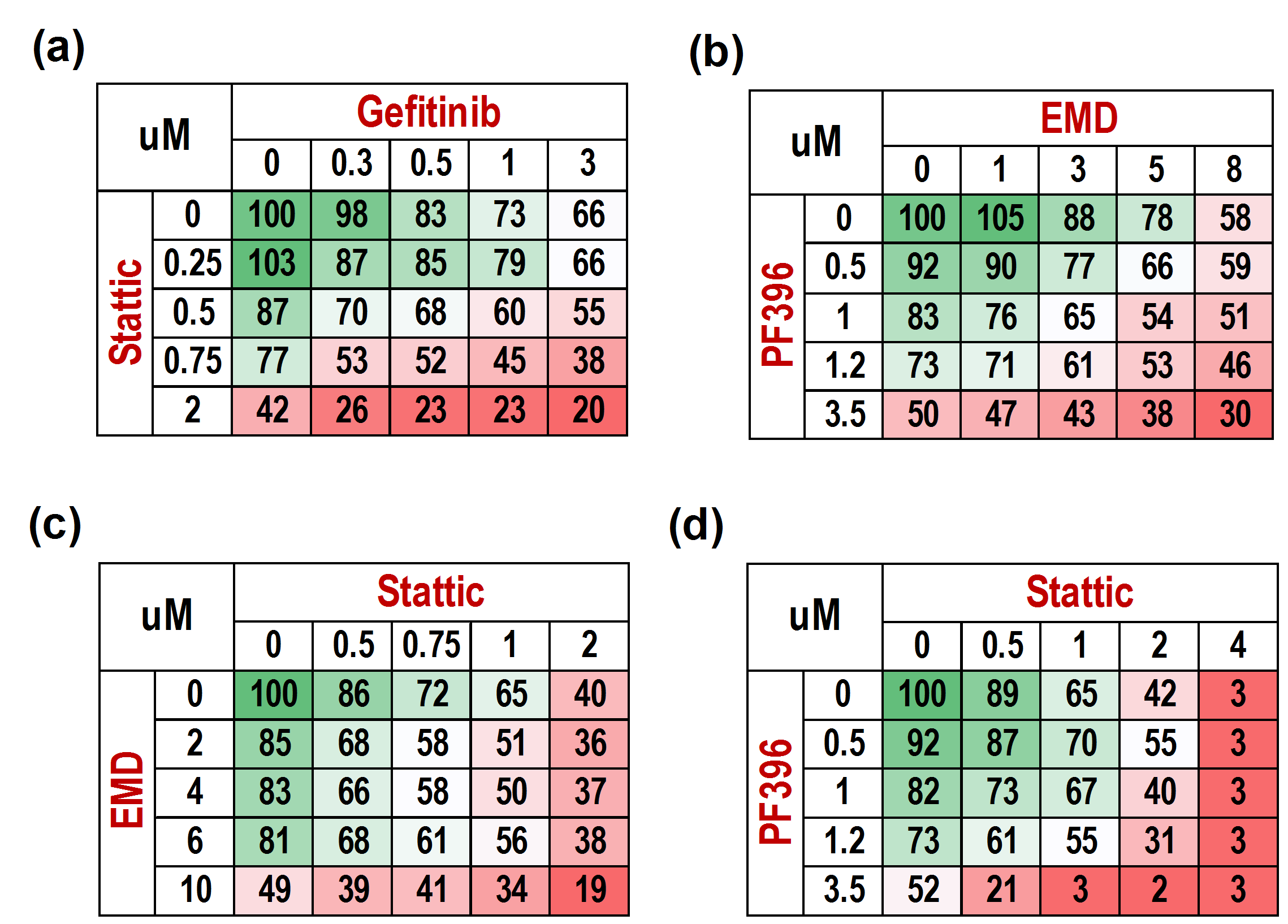

Supplement: S8 Fig — Inhibition effects on cell growth are depicted as percentage relative to non-treated cells. Colour gradient code: from untreated (green) to strongly inhibited (red). (TIF) [file pcbi.1006192.s011.tif]

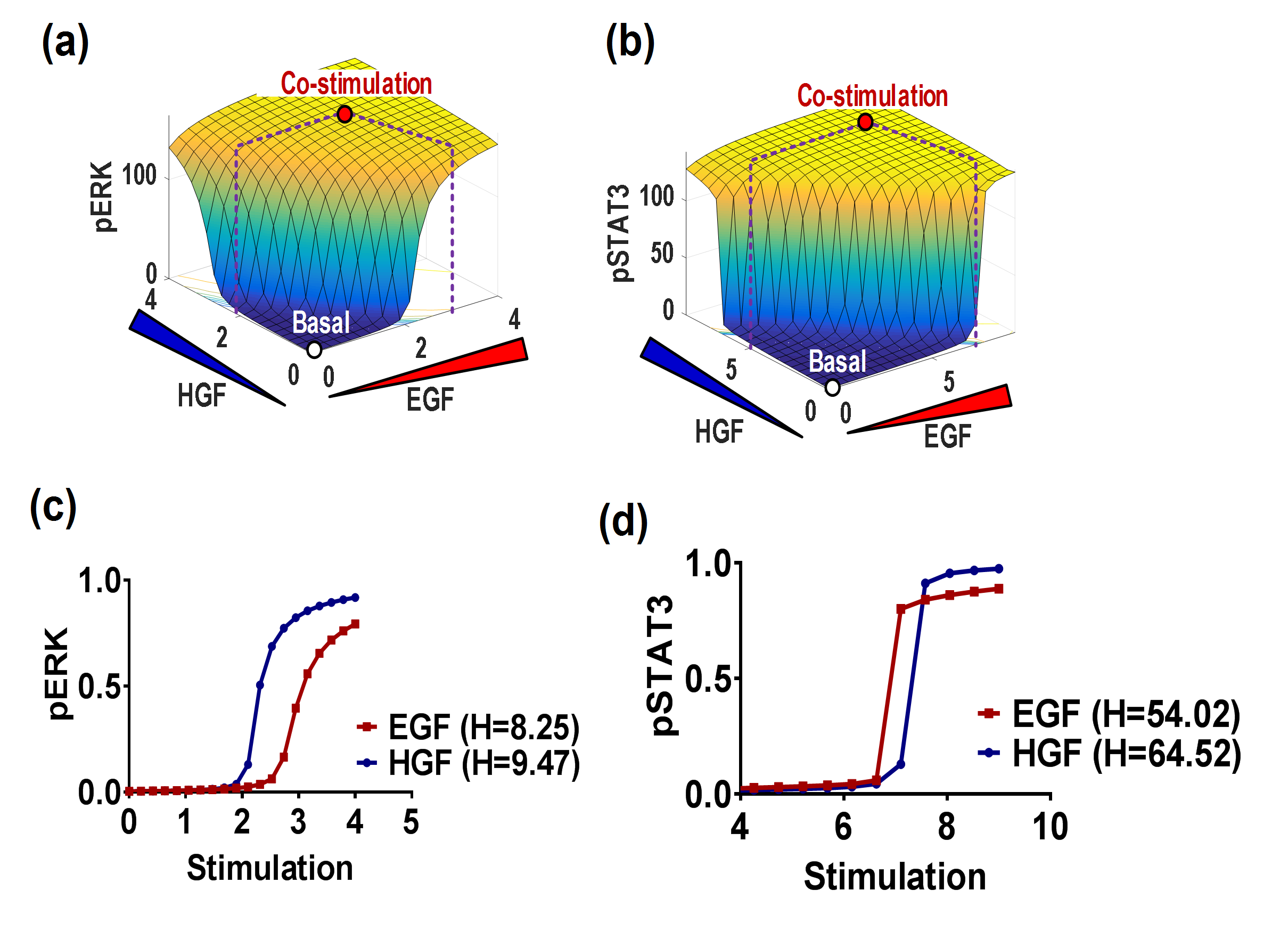

Supplement: S9 Fig — (a-b) 3-dimensional (3D) simulations of the (normalised) steady-state pERK/pSTAT3 levels to simultaneous increases in EGF and HGF stimulation, displaying an switch-like pattern. (c-d) 2D dose-response simulations of steady-state pERK/pSTAT3 to Gefitinib and PF396, respectively. H denotes the fitted Hill coefficient of the dose response curve. (TIF) [file pcbi.1006192.s012.tif]

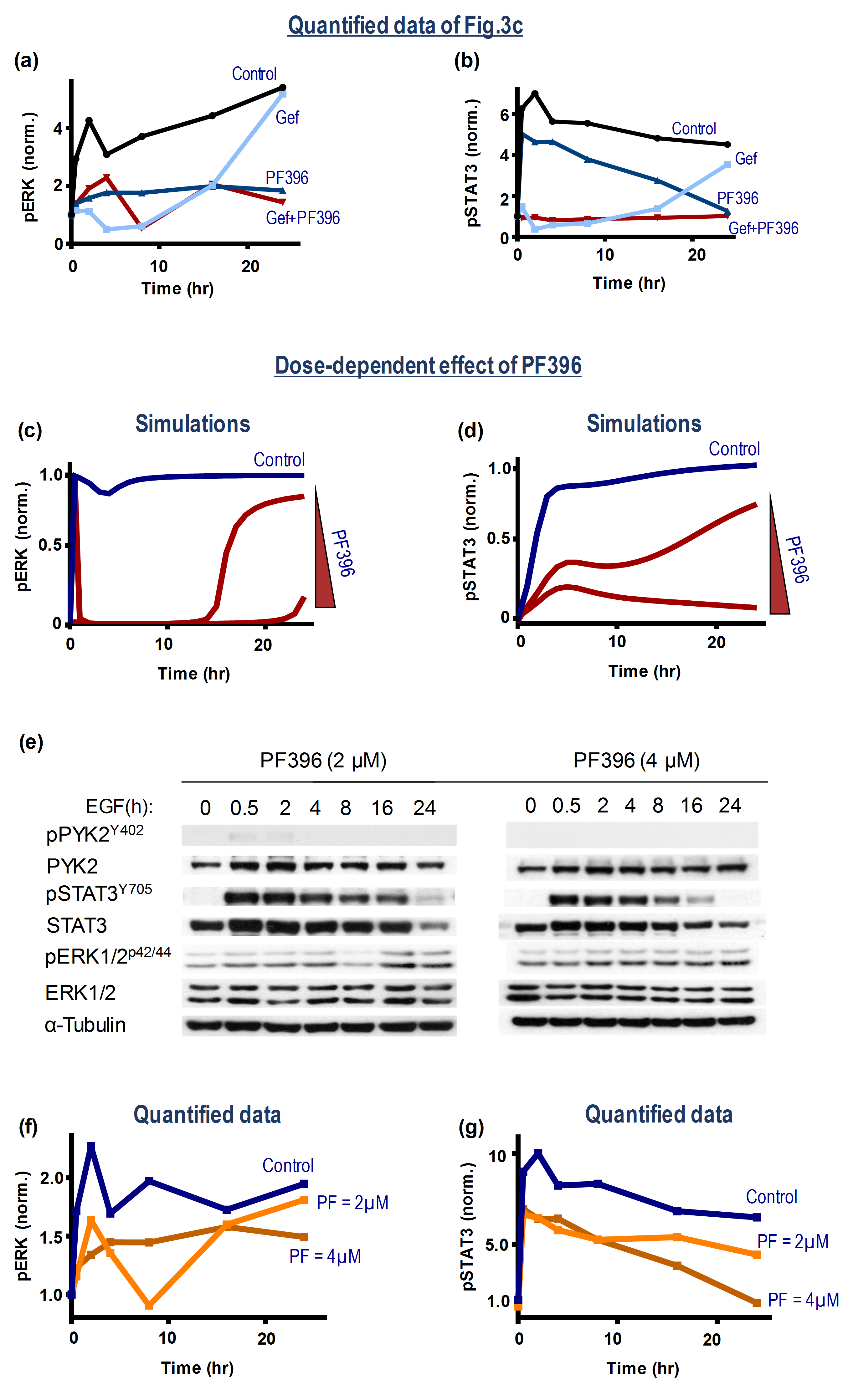

Supplement: S10 Fig — (a-b) Quantification of Western blot data in Fig 3C, main text. (c-d) Simulated time profile of pERK and pSTAT3. Model simulations show dose-dependent effect of PF396-mediated PYK2 inhibition on the response of STAT3 and ERK activities. (e) Western blot data measuring signalling responses to increasing doses of PF396. (f-g) Quantification of the data in (e), control was the same as in Fig 3C, main text. (TIF) [file pcbi.1006192.s013.tif]

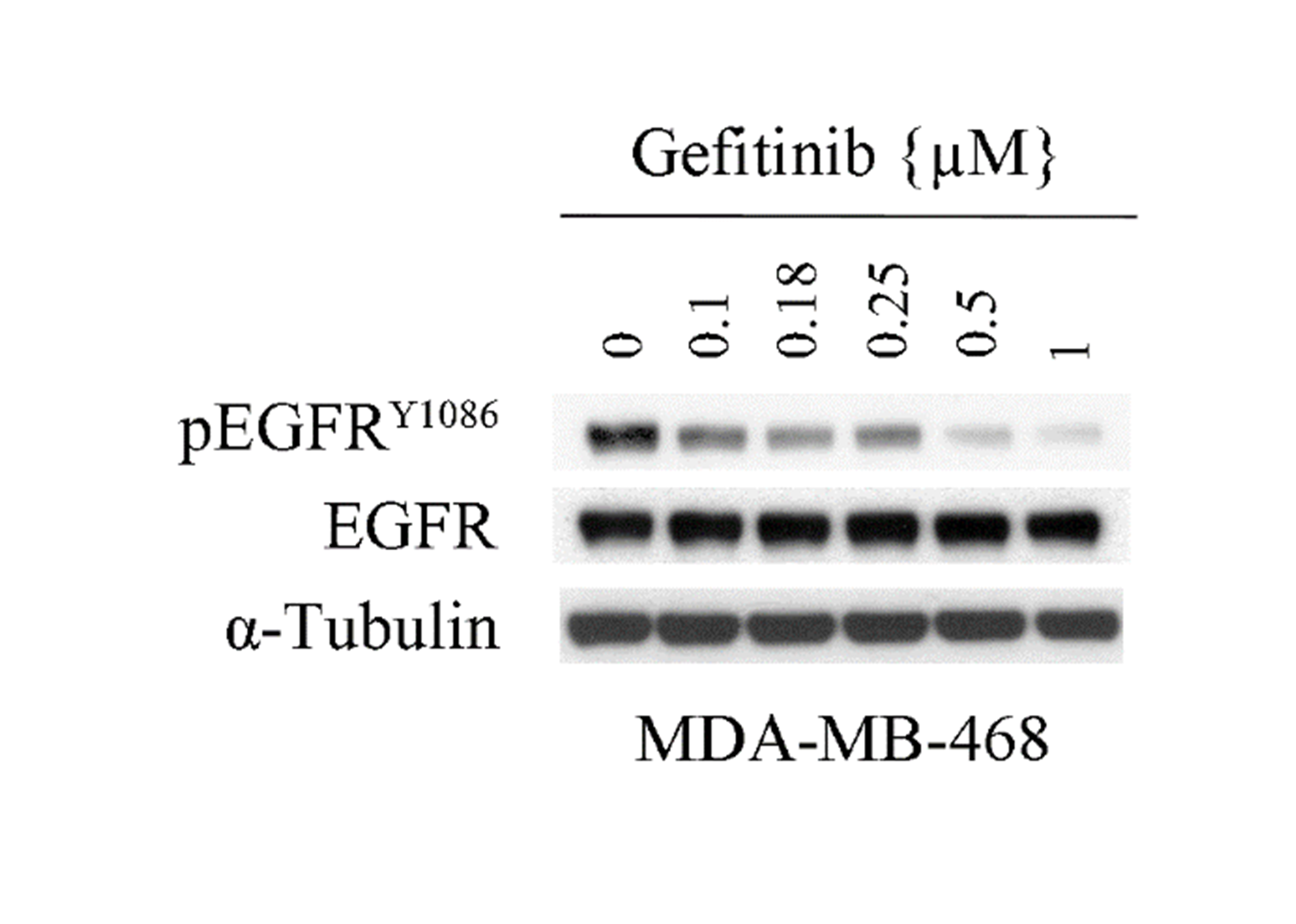

Supplement: S11 Fig — To find the lowest usable concentration of Gefitinib to inhibit EGFR phosphorylation, cells were treated with gradually increasing concentrations of the inhibitor for 24 hrs. Phosphorylated and total protein levels were assayed by Western Blot. (TIF) [file pcbi.1006192.s014.tif]

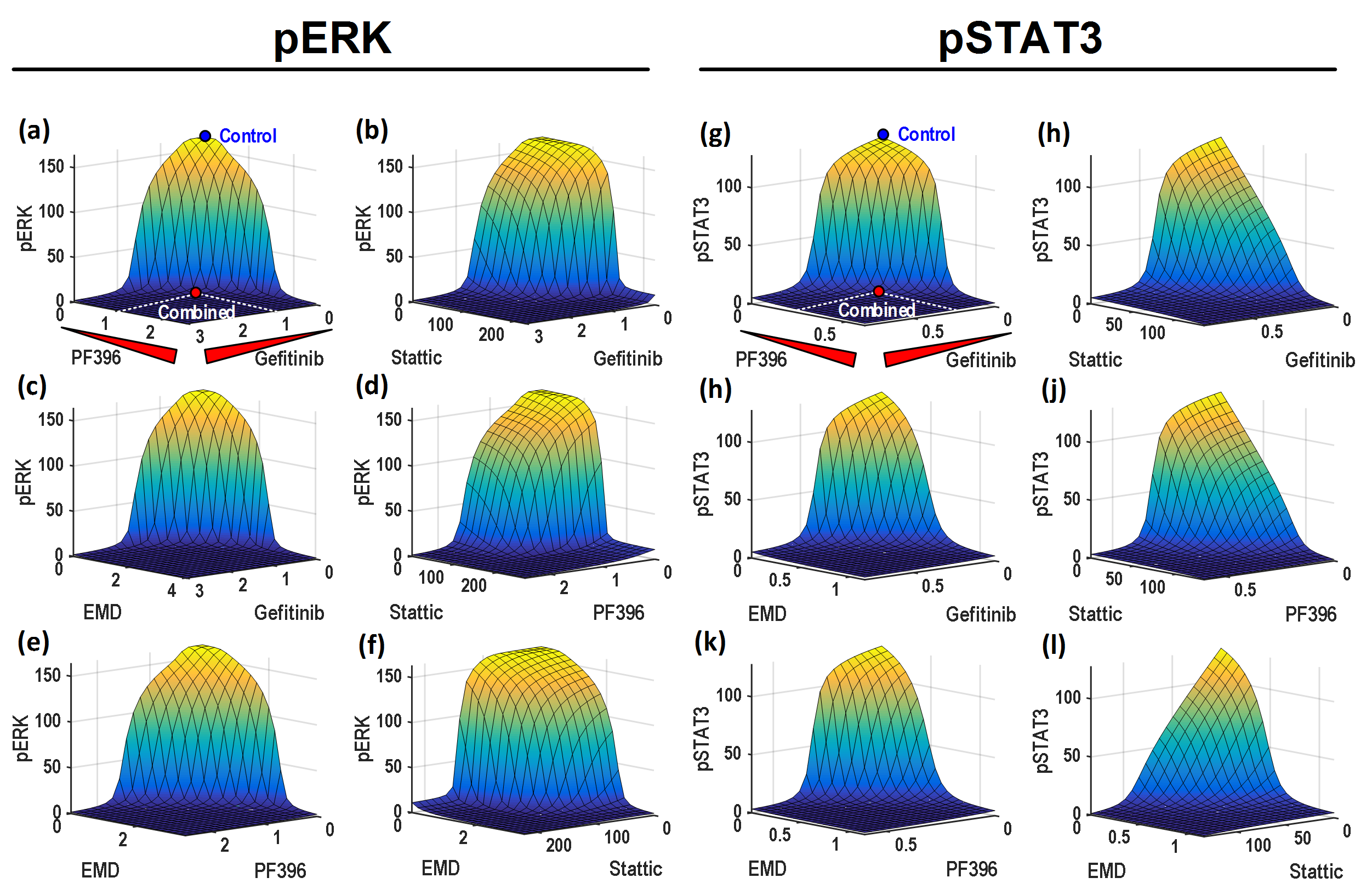

Supplement: S12 Fig — (TIF) [file pcbi.1006192.s015.tif]

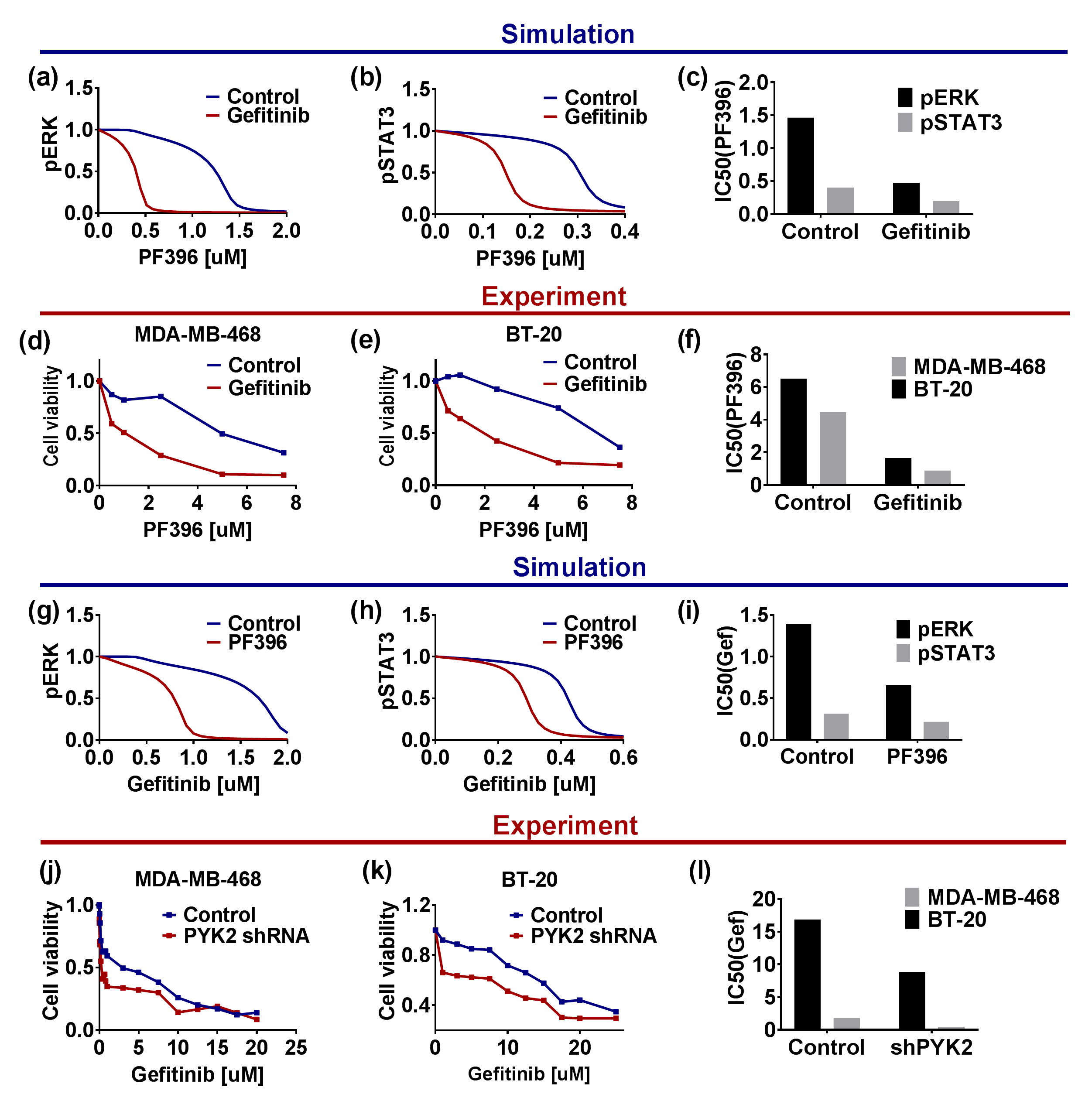

Supplement: S13 Fig — (a,b) Simulated dose-response of pERK/pSTAT3 to increasing PF396 in the absence or presence of Gefitinib pre-treatment, showing Gefitinib increased cancer cell sensitivity to PF396. (c) Quantified IC50 values of PF396 in the absence or presence of Gefitinib pre-treatment. (d,e) Cell viability in response to PF-396 in the absence or presence of Gefitinib, measured in MDA-MB-468 and BT-20 cells [12]. Pre-treatment with Gefitinib significantly increased drug sensitivity to PF396 as predicted. (f) Quantified IC50 values of PF396 in the absence or presence of Gefitinib pre-treatment. (g,h) Simulated dose-response of pERK/pSTAT3 to increasing Gefitinib in the absence or presence of PF396 treatment, which sensitised cancer cells to Gefitinib. (i) Quantified IC50 values of Gefitinib in the absence or presence of PF396 treatment. (j,k) Cell viability in response to Gefitinib in normal or PYK2-deficient cells measured in MDA-MB-468 and BT-20 cells by colony formation assay [12]. Combined treatment with PF396 significantly increased drug sensitivity to Gefitinib. (l) Quantified IC50 values of Gefitinib in the absence or presence of PF396 treatment. (TIF) [file pcbi.1006192.s016.tif]

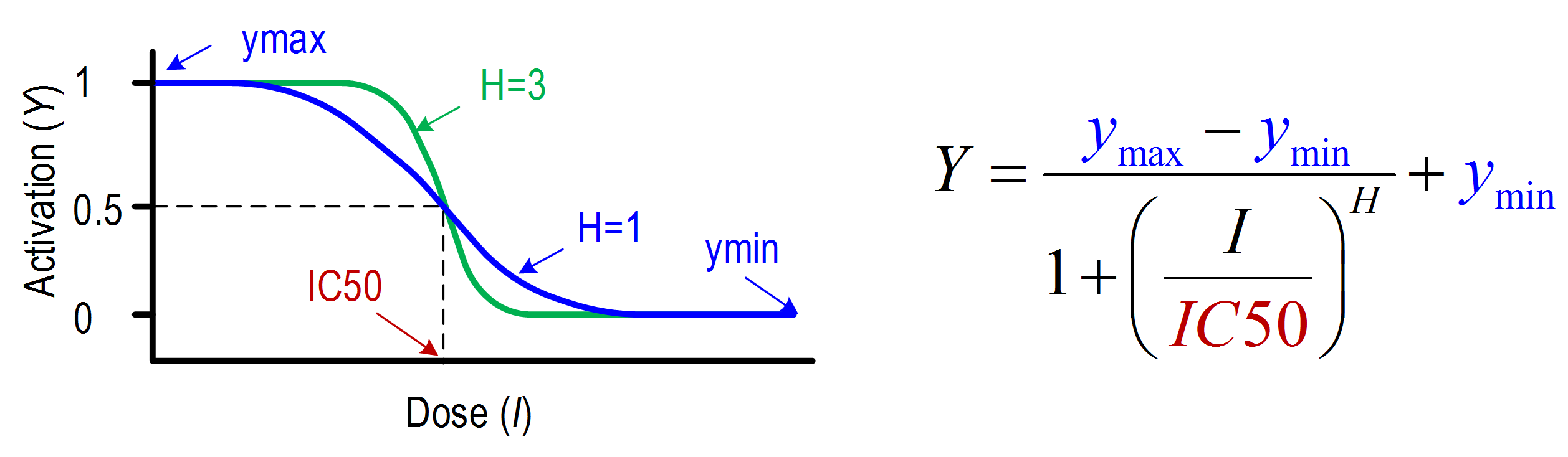

Supplement: S14 Fig — The fitted Hill coefficient (H) signifies the switch-ness (steepness) of the response curve: a higher H value shows that the response curve is more switch-like (steep). ymax and ymin are the maximum and minimum value of the output response. On the other hand, IC50 denotes the half-maximal concentration and signifies the sensitivity of the output to the inhibitor (I). (TIF) [file pcbi.1006192.s017.tif]

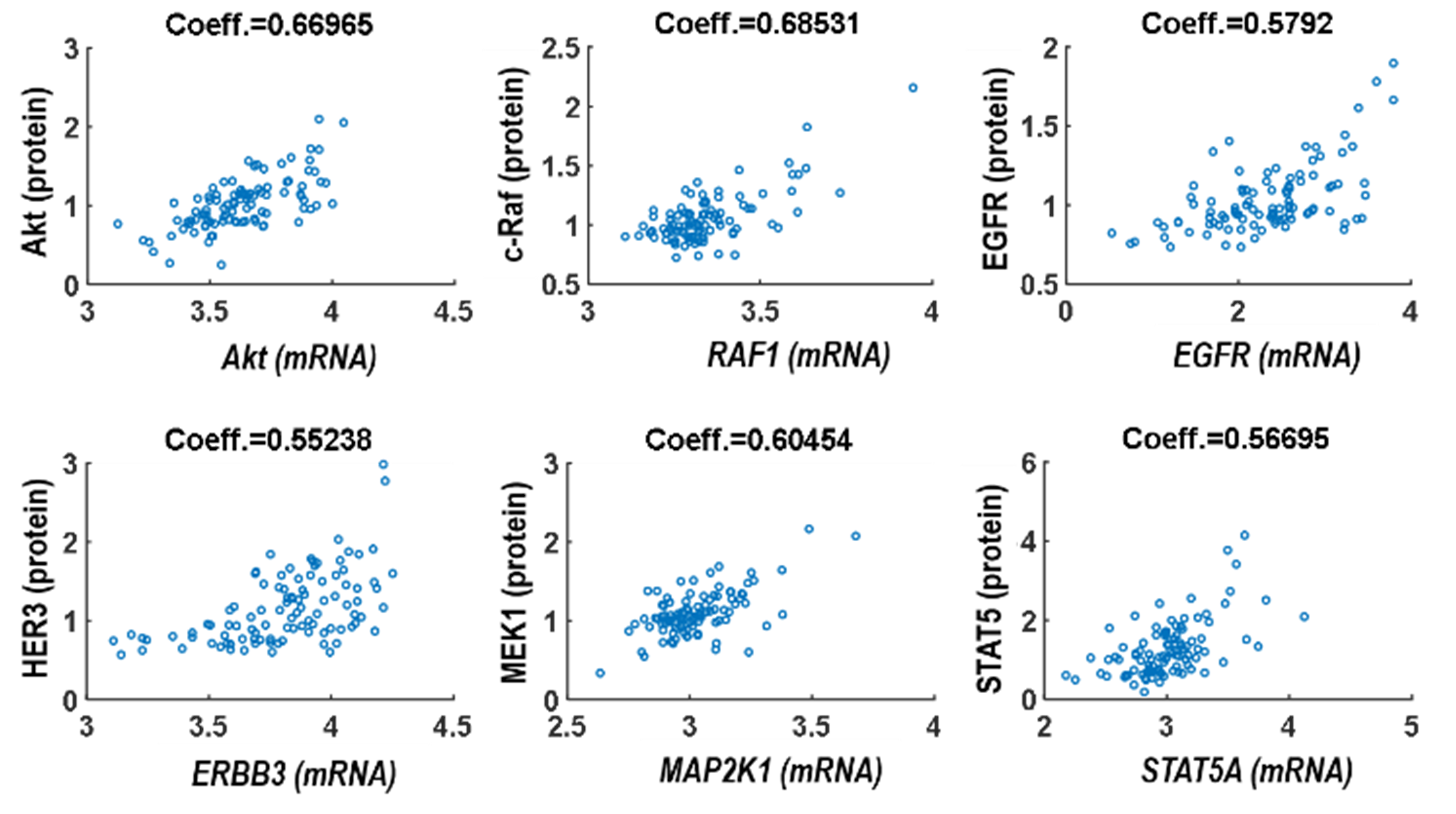

Supplement: S15 Fig — Correlation analysis of >1000 BC patients from TCGA [39] revealed relatively strong correlations between protein and RNA expression levels of signalling molecules that have both types of data available, suggesting RNA levels can serve as a good proxy for protein expression. (TIF) [file pcbi.1006192.s018.tif]

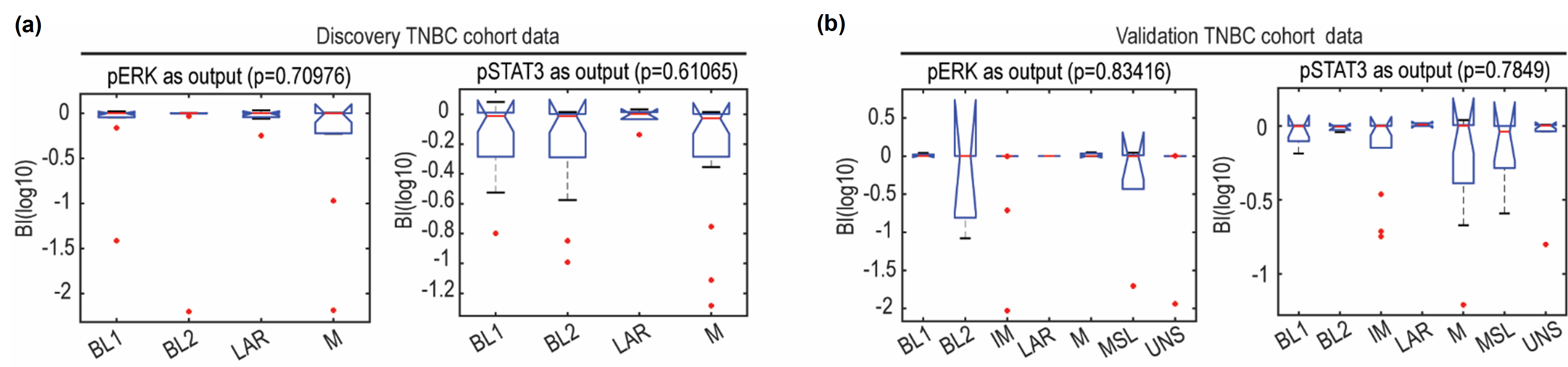

Supplement: S16 Fig — The discovery (a) and validation cohort (b) datasets both show no significant differences in the predicted synergy index (assessed by BI scores) between the patients classified using the Vanderbilt-based subtyping system. The computed synergy indexes for Gefitinib + PF396 co-treatment of statistical significance were calculated based on analysis of variance (ANOVA). (TIF) [file pcbi.1006192.s019.tif]

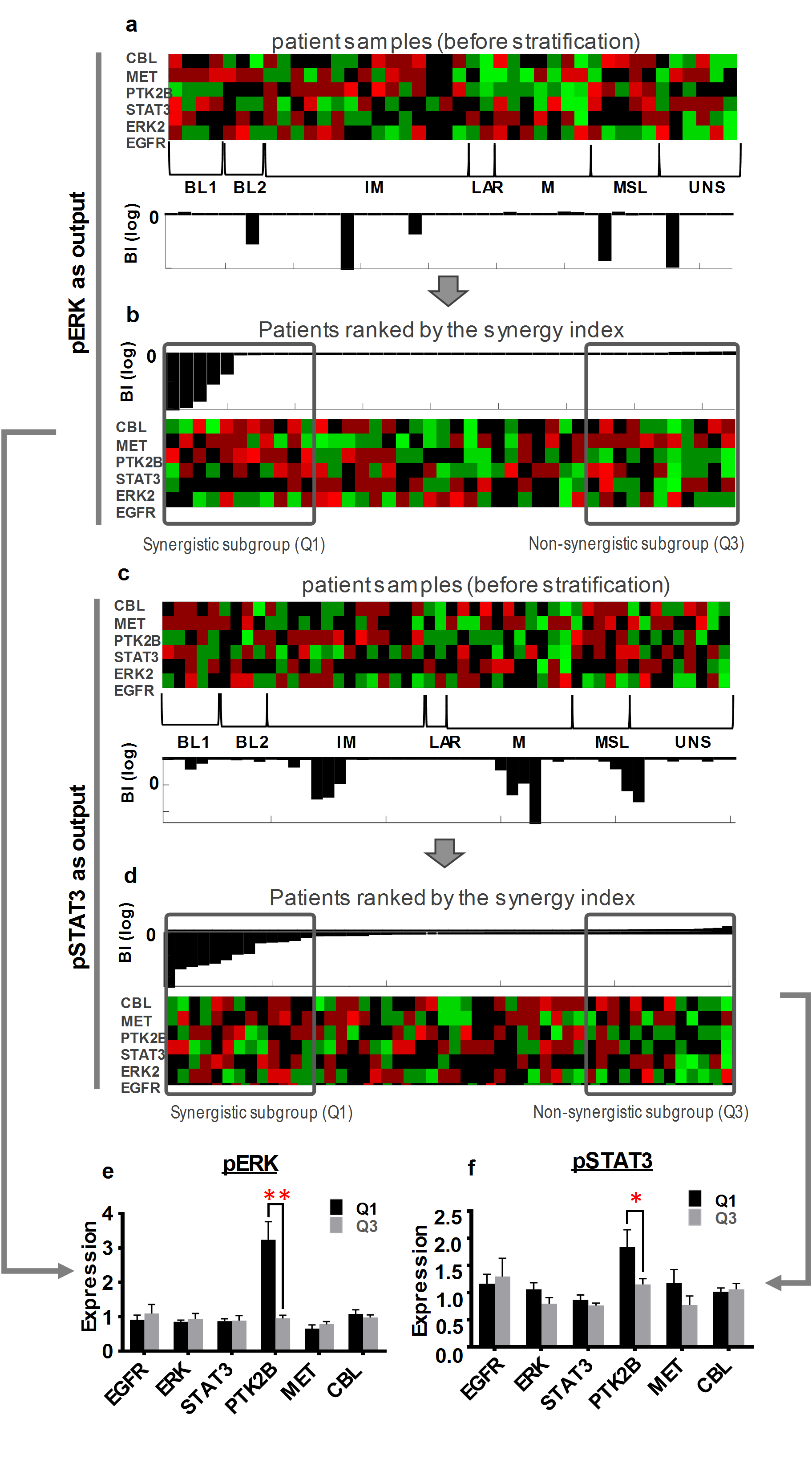

Supplement: S17 Fig — The validation TNBC cohort data were first clustered into Vanderbilt-based subtypes (upper panel). Synergy indices (BI scores) of EGFR and PYK2 co-inhibition predicted for each patient using the patient-specific network model (lower panels) are depicted. Only patients displaying sensitivity to Gefitinib and PF396 alone are included for synergy analysis for the combined treatment (i.e. 42 and 50 patients for pERK and pSTAT3 as readouts, respectively). IC50 concentrations were used for each drug. (a-b) Stratification analysis with pERK as readout. Patient samples sorted by the predicted synergistic effect. Q1 and Q3 denote the ‘synergistic’ and ‘antagonistic’ patient subgroups, respectively. Q1 and Q3 contain patients having synergy index values in the lower quartile and in the upper quartile, respectively. (c-d) Stratification analysis with pSTAT3 as readout as in panels (a-b). (e-f) Differential gene expression analysis between the Q1 and Q3 subgroups, showing upregulation of PYK2 is statistically significant in Q1 subgroup for both signalling readouts. *P<0.05; **P<0.01. (TIF) [file pcbi.1006192.s020.tif]

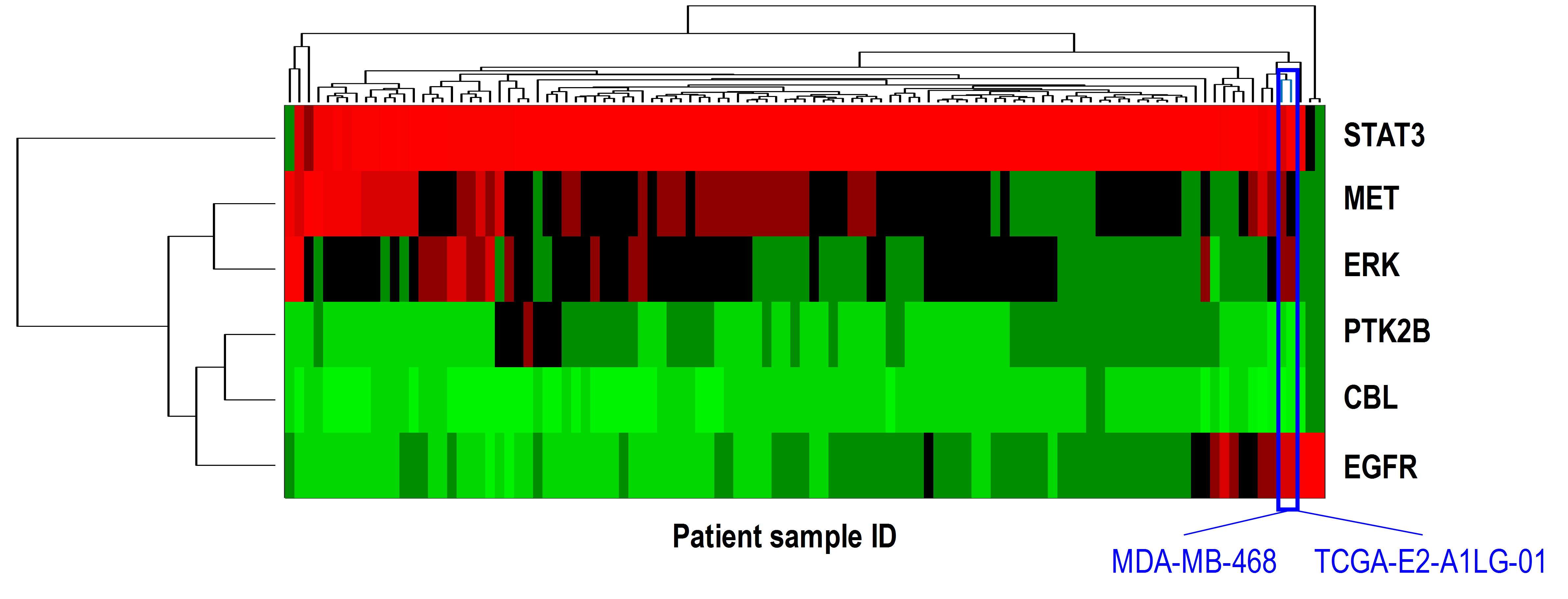

Supplement: S18 Fig — Using gene expression data, hierarchical clustering method identified that the expression profile of the sample ID TCGA-E2-A1LG-01 within the discovery cohort is most similar to that of the MDA-MB-468 cell line. This patient then serves as a reference for normalising data as model inputs to generate patient-specific models. (TIF) [file pcbi.1006192.s021.tif]

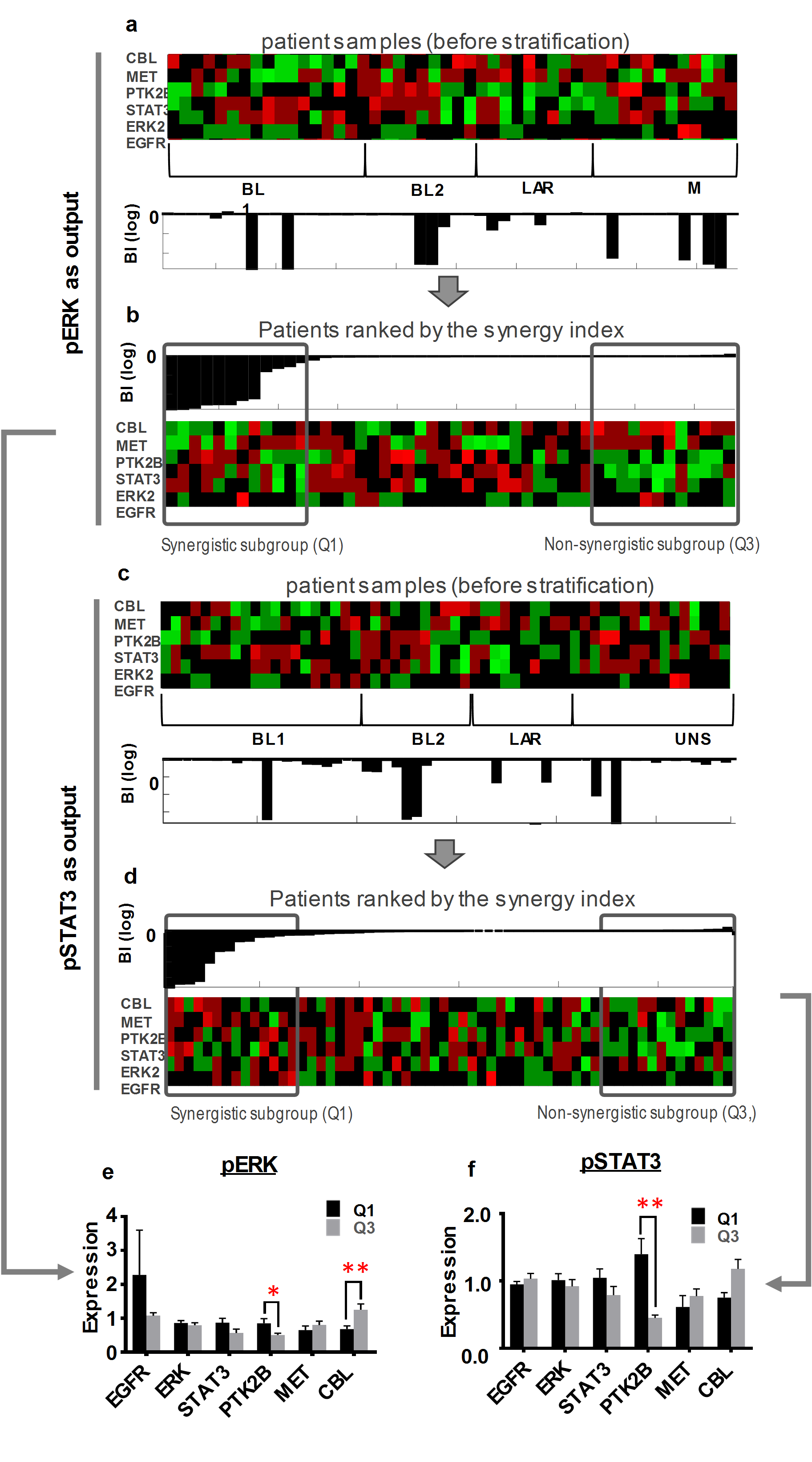

Supplement: S19 Fig — The discovery TNBC cohort data were normalized by the patient sample whose molecular profile is most similar to that of the MDA-MB-468 cell line using hierarchical clustering method (S18 Fig). The normalized patient samples were first clustered into four Vanderbilt based subtypes. Synergy indices (BI scores) of EGFR and PYK2 co-inhibition predicted for each patient using the patient-specific network model (lower panels) are depicted. Only patients displaying sensitivity to Gefitinib and PF396 alone are included for synergy analysis in combined treatment (i.e. 48 and 57 patients for pERK and pSTAT3 as readouts, respectively). IC50 concentrations were used for each drug. (a-b) Stratification analysis with pERK as readout. Patient samples sorted by the predicted synergistic effect. Q1 and Q3 denote the ‘synergistic’ and ‘antagonistic’ patient subgroups, respectively. Q1 and Q3 contain patients having synergy index values in the lower quartile and in the upper quartile, respectively. (c-d) Stratification analysis with pSTAT3 as readout as in panels (a-b). (e-f) Differential gene expression analysis between the Q1 and Q3 subgroups, showing upregulation of PYK2 is statistically significant in Q1 subgroup for both signalling readouts. *P<0.05; **P<0.01. (TIF) [file pcbi.1006192.s022.tif]

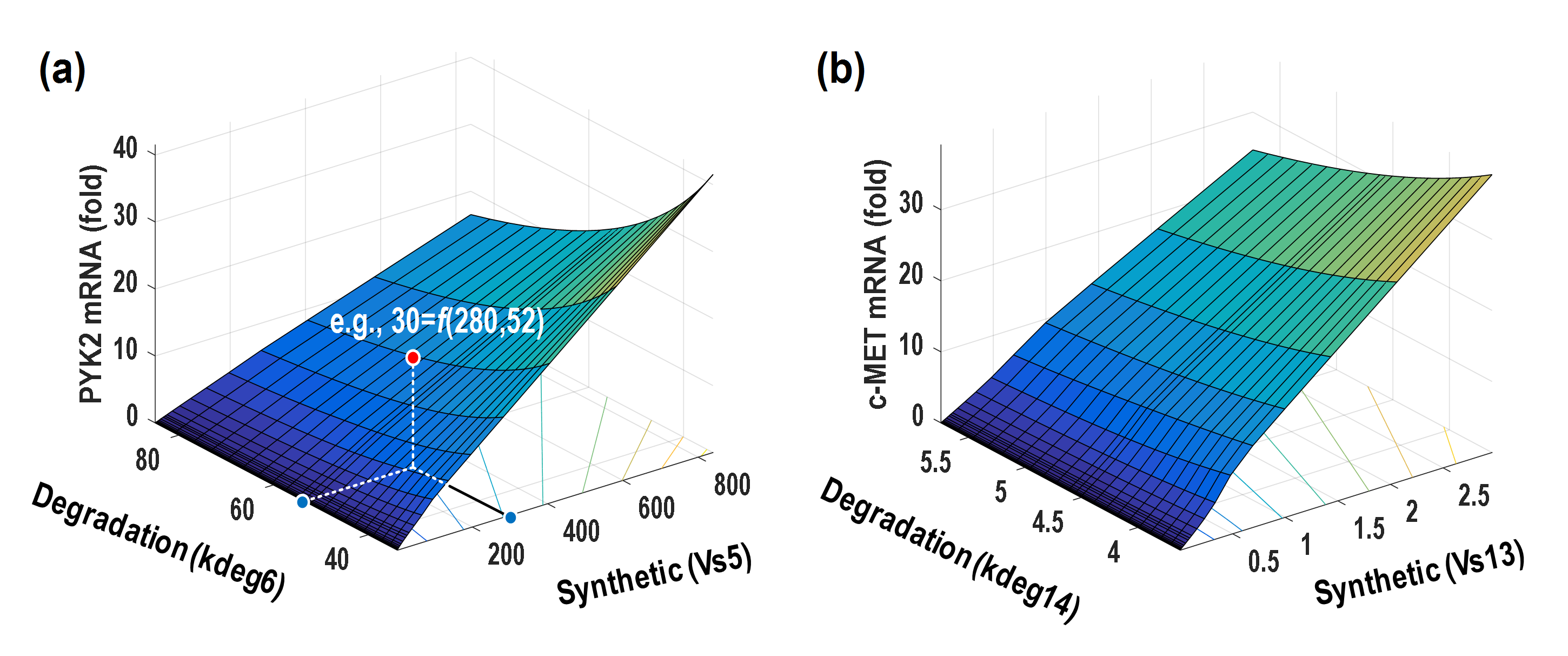

Supplement: S20 Fig — Implicit reverse functions used for adjustment of kinetic parameter values of PYK2 (a) and c-Met mRNA (b). In order to generate patient specific network model, we adjusted mRNA levels of PYK2 and c-Met by modifying kinetic parameter values (Vs5 and kdeg6 for PYK2 mRNA, and Vs13 and kdeg14 for c-Met mRNA) based on implicit reverse functions. For example, if the tumour sample showed a 30 fold increase in PYK2 gene expression, then we modified kinetic parameter values of kdeg6 = 280 and Vs5 = 52 based on the implicit reverse function. (TIF) [file pcbi.1006192.s023.tif]
