# Supplementary material for: Systems modelling of the EGFR-PYK2-c-Met interaction network predicts and prioritizes synergistic drug combinations for triple-negative breast cancer
Source: PLoS Comput Biol. 2018 Jun 19;14(6):e1006192. doi: 10.1371/journal.pcbi.1006192 (PMC6007894; doi:10.1371/journal.pcbi.1006192)
Supplement: S1 Table — (DOCX) [file pcbi.1006192.s024.docx]

**Table S1. Reactions and reaction rates of the EGFR-c-MET-PYK2 network model**

|  | **Reaction** | **Reaction rates** |
| --- | --- | --- |
| v1 | EGFR → pEGFR | kc1*(EGF/(1+Gefitinib/Ki1)+caEGF)*EGFR/(Km1+EGFR) |
| v2 | pEGFR → EGFR | (Vmax2+kc2*aPTP)*pEGFR/(Km2+pEGFR) |
| v3 | EGFR → EGFRub | (Vmax3+kc3*pCbl)*EGFR/(Km3+EGFR)*Ki3a/(Ki3a+PYK2tot/(1+PF396/Ki3b)) |
| v4 | EGFRub → EGFR | Vmax4*EGFRub/(Km4+EGFRub) |
| v5 | ∅ → PYK2m | Vs5 + Vmax5*pSTAT3/(Km5+pSTAT3) |
| v6 | PYK2m → ∅ | kdeg6*PYK2m |
| v7 | PYK2m → PYK2 | Vmax7*PYK2m/(Km7+PYK2m) |
| v8 | PYK2 → ∅ | kdeg8*PYK2 |
| v9 | PYK2 → pPYK2 | (kc9a*pEGFR+kc9b*pcMET/(1+EMD/Ki9))*PYK2/(Km9+PYK2) |
| v10 | pPYK2 → PYK2 | (Vmax10+kc10*aPTP)*pPYK2/(Km10+pPYK2) |
| v11 | STAT3→ pSTAT3 | kc11*(pPYK2/(1+PF396/Ki3b))*STAT3/(Km11+STAT3) |
| v12 | pSTAT3→ STAT3 | (Vmax12+kc12*aPTP)*pSTAT3/(Km12+pSTAT3) |
| v13 | ∅ → cMETm | Vs13 + Vmax13*pSTAT3/(Km13+pSTAT3) |
| v14 | cMETm→ ∅ | kdeg14*cMETm |
| v15 | cMETm → cMET | Vmax15*cMETm/(Km15+cMETm) |
| v16 | cMET → ∅ | (kdeg16+kc16*pCbl)*cMET/(Km16+cMET) |
| v17 | cMET → pcMET | (kc17*HGF+caHGF)*cMET/(Km17+cMET) |
| v18 | pcMET → cMET | Vmax18*pcMET/(Km18+pcMET) |
| v19 | Cbl → pCbl | kc19*pEGFR*Cbl/(Km19+Cbl) |
| v20 | pCbl → Cbl | (Vmax20+kc20*aPTP)*pCbl/(Km20+pCbl) |
| v21 | PTP → aPTP | kc21*pEGFR*PTP/(Km21+PTP) |
| v22 | aPTP → PTP | Vmax22*aPTP/(Km22+aPTP) |
| v23 | ERK → pERK | (kc23a*pcMET/(1+EMD/Ki23)+kc23b*pEGFR)*ERK/(Km23+ERK) |
| v24 | pERK → ERK | Vmax24*pERK/(Km24+pERK) |
| v25 | STAT3 + Stattic  ↔ STAT3uStattic | ka25*STAT3*Stattic - kd25*STAT3uStattic |

*Footnote:* The effects of the inhibitors Gefitinib, PF396 and EMD were modelled by incorporating into the rate equations v1, v3 & v11 and v9 & v23, respectively. We assumed that Gefitinib inhibits EGFR phosphorylation since Gefitinib effectively inhibits all EGFR tyrosine phosphorylation sites in both high and low EGFR-expressing cell lines [1]. Parameter Ki1 represents the inhibition strength exerted by Gefitinib on EGFR. PYK2 inhibitor PF396 is a kinase inhibitor that blocks the transferring a phosphate group to a target protein from ATP, and it directly binds to the active site of PYK2 and inhibits its function [2]. Thus, PF396 was assumed to inhibit the kinase activity of PYK2, and its strength is indicated by the parameter Ki3b (while Ki3a is a kinetic parameter value associated with PYK2 inhibition of EGFR ubiquitination). We assumed that the c-Met inhibitor EMD-1214063 (EMD) is an ATP-competitive small molecule that inhibits c-Met phosphorylation of target substrates [3,4]. Ki9 and Ki23 represent inhibition coefficients of the c-Met inhibitor towards PYK2 and ERK as c-Met substrates, respectively. Furthermore, the effect of the STAT inhibitor, Stattic was modelled according to a reversible drug-target binding reaction (as in v25) since Stattic was reported to selectively inhibit dimerization and activation of STAT3 [5]. We assumed that the STAT3 inhibitor directly bind and form an inhibition complex and Ka25 and Kd25 denotes the binding coefficients. The binding affinity (kd25/ka25) was estimated 92 µM based on the previous experimental observation [5]. They displayed the STAT3 activation was fully suppressed around 200-250µM and the half-maximal concentration was about 100µM. Ki3a was estimated based on the time-course training data shown in Fig 1c-g. Ki3b were estimated based on the PYK2 perturbation data in Fig 1o-r. Ki9 and Ki23 were estimated using c-Met inhibition data used for training now shown in Fig. S1. As we did not use Gefitinib related data to train the model, Ki1 was set to a fixed value. Note that variation of Gefitinib- and PF396- associated parameters (Ki1 and Ki3b) does not have any significant influence on the CI scores of drug synergy (Fig. S5).

**SUPPLEMENTARY REFERENCE**

1. Pedersen MW, Pedersen N, Ottesen LH, Poulsen HS (2005) Differential response to gefitinib of cells expressing normal EGFR and the mutant EGFRvIII. British Journal of Cancer 93: 915-923.

2. Han S, Mistry A, Chang JS, Cunningham D, Griffor M, et al. (2009) Structural Characterization of Proline-rich Tyrosine Kinase 2 (PYK2) Reveals a Unique (DFG-out) Conformation and Enables Inhibitor Design. The Journal of biological chemistry 284: 13193-13201.

3. Dussault I, Bellon SF (2009) From concept to reality: the long road to c-Met and RON receptor tyrosine kinase inhibitors for the treatment of cancer. Anti-cancer agents in medicinal chemistry 9: 221-229.

4. Allen JV, Bardelle C, Blades K, Buttar D, Chapman L, et al. (2011) The discovery of benzanilides as c-Met receptor tyrosine kinase inhibitors by a directed screening approach. Bioorganic & Medicinal Chemistry Letters 21: 5224-5229.

5. Schust J, Sperl B, Hollis A, Mayer TU, Berg T (2006) Stattic: A Small-Molecule Inhibitor of STAT3 Activation and Dimerization. Chemistry & Biology 13: 1235-1242.
