# Supplementary material for: Systems modelling of the EGFR-PYK2-c-Met interaction network predicts and prioritizes synergistic drug combinations for triple-negative breast cancer
Source: PLoS Comput Biol. 2018 Jun 19;14(6):e1006192. doi: 10.1371/journal.pcbi.1006192 (PMC6007894; doi:10.1371/journal.pcbi.1006192)
Supplement: S2 Table — The reaction rates are given in S1 Table. (DOCX) [file pcbi.1006192.s025.docx]

**Table S2. Ordinary differential equations of the EGFR-c-MET-PYK2 model.** The reaction rates are given in Supplementary Table 1.

| **Left-hand Sides** | **Right-hand Sides** | **Initial Conditions (nM)** |
| --- | --- | --- |
| d[pEGFR]/dt | v1 - v2 | 0.109 |
| d[EGFRub]/dt | v3 - v4 | 6.940 |
| d[PYK2m]/dt | v5 – v6 | 0.622 |
| d[PYK2]/dt | v7 - v8 - v9 + v10 | 9.299 |
| d[pPYK2]/dt | v9 - v10 | 2.510 |
| d[pSTAT3]/dt | v11 - v12 | 1.178 |
| d[cMETm]/dt | v13 - v14 | 0.023 |
| d[cMET]/dt | v15 - v16 - v17 + v18 | 4.672 |
| d[pcMET]/dt | v17 - v18 | 0.502 |
| d[pCbl]/dt | v19 - v20 | 10.476 |
| d[aPTP]/dt | v21 - v22 | 0.494 |
| d[pERK]/dt | v23 - v24 | 0.669 |
| d[STAT3uStattic]/dt | v25 | 0.00 |

Due to the law of conversation, the other model state variables could be calculated using the following algebraic equations:

EGFR(t) = EGFR_tot_ – pEGFR(t) – EGFRub(t),

STAT3(t)=STAT3_tot_ - pSTAT3(t) -STAT3uStattic(t),

Cbl(t)=Cbl_tot_ – pCbl(t),

PTP(t)=PTP_tot_ – aPTP(t),

ERK(t)=ERK_tot_ – pERK(t) and

Stattic(t) = Stattic_tot_ - STAT3uStattic(t),

where EGFR_tot_, STAT3_tot_, Cbl_tot_, PTP_tot_, ERK_tot_ and Stattic_tot_ denote the total concentrations of the corresponding molecules.
