# Supplementary material for: Systems modelling of the EGFR-PYK2-c-Met interaction network predicts and prioritizes synergistic drug combinations for triple-negative breast cancer
Source: PLoS Comput Biol. 2018 Jun 19;14(6):e1006192. doi: 10.1371/journal.pcbi.1006192 (PMC6007894; doi:10.1371/journal.pcbi.1006192)
Supplement: S3 Table — (DOCX) [file pcbi.1006192.s026.docx]

**Table S3. Best-fitted parameter values used for simulations.**

| **Parameter** | **Value** | **Unit** |
| --- | --- | --- |
| kc1 | 413.0475 | min^-1^ |
| Km1 | 248.8857 | nM |
| Ki1 | 1.0000 | µM |
| kc2 | 1406.048 | min^-1^ |
| Km2 | 3.801894 | nM |
| Vmax3 | 0.000104 | nM min^-1^ |
| Km3 | 2.285599 | nM |
| Ki3a | 0.08356 | nM |
| Ki3b | 1.0000 | nM |
| Vmax4 | 11.11732 | nM min^-1^ |
| kc3 | 10.78947 | min^-1^ |
| Km4 | 90.78205 | nM |
| Vs5 | 26.54606 | nM min^-1^ |
| Vmax5 | 34.04082 | nM min^-1^ |
| Km5 | 4.74242 | nM |
| kdeg6 | 53.57967 | min^-1^ |
| Vmax7 | 3.349654 | nM min^-1^ |
| Km7 | 3.334264 | nM |
| kc9a | 0.463447 | min^-1^ |
| kc9b | 0.988553 | min^-1^ |
| Km9 | 34.91403 | nM |
| Vmax10 | 0.530884 | nM min^-1^ |
| Km10 | 9.141132 | nM |
| kdeg8 | 0.056624 | min^-1^ |
| kc11 | 0.321366 | min^-1^ |
| Km11 | 20.6063 | nM |
| kc12 | 0.00029 | min^-1^ |
| Km12 | 11.58777 | nM |
| Vs13 | 0.093756 | nM min^-1^ |
| Vmax13 | 0.354813 | nM min^-1^ |
| Km13 | 38.72576 | nM |
| kdeg14 | 4.560369 | min^-1^ |
| Vmax15 | 91.41132 | nM min^-1^ |
| Km15 | 6.456542 | nM |
| kc17 | 0.000811 | min^-1^ |
| Km17 | 9.817479 | nM |
| Vmax18 | 0.060674 | nM min^-1^ |
| Km18 | 9.954054 | nM |
| kdeg16 | 24.49063 | min^-1^ |
| kc16 | 1.174898 | min^-1^ |
| Km16 | 528.4453 | nM |
| kc19 | 52.72299 | min^-1^ |
| Km19 | 13.30454 | nM |
| kc20 | 35.64511 | min^-1^ |
| Km20 | 24.32204 | nM |
| kc21 | 0.003972 | min^-1^ |
| Km21 | 52.72299 | nM |
| Vmax22 | 0.034914 | nM min^-1^ |
| Km22 | 46.45153 | nM |
| Vmax2 | 112.2018 | nM min^-1^ |
| Vmax12 | 7.638358 | nM min^-1^ |
| Vmax20 | 0.048306 | nM min^-1^ |
| kc23a | 7.03E+09 | min^-1^ |
| kc23b | 8.43E+08 | min^-1^ |
| Km23 | 2.831392 | nM |
| Vmax24 | 4.4E+09 | nM min^-1^ |
| Km24 | 0.156675 | nM |
| kc10 | 0.006109 | min^-1^ |
| Ki9 | 1.65577 | nM |
| Ki23 | 13.48963 | nM |
| ka25 | 127.3503 | µM^-1^ min^-1^ |
| kd25 | 11.74898 | min^-1^ |
| caEGF | 0.089125 | nM |
| caHGF | 0.009036 | nM |
| EGFRtot | 398.1072 | nM |
| STAT3tot | 144.2115 | nM |
| Cbltot | 174.9847 | nM |
| PTPtot | 296.4831 | nM |
| ERKtot | 166.7247 | nM |
