# Supplementary material for: Systems modelling of the EGFR-PYK2-c-Met interaction network predicts and prioritizes synergistic drug combinations for triple-negative breast cancer
Source: PLoS Comput Biol. 2018 Jun 19;14(6):e1006192. doi: 10.1371/journal.pcbi.1006192 (PMC6007894; doi:10.1371/journal.pcbi.1006192)
Supplement: S4 Table — (DOCX) [file pcbi.1006192.s027.docx]

**Table S4. Parameters perturbed for feedback functional analysis shown in Fig. 3h-o, Fig. 5d-o.**

| **Feedback Link** | **Parameter** | **Interaction** |
| --- | --- | --- |
| F1 | Ki3a | PYK2 inhibition of EGFR degration |
| F2 | Vmax5 | STAT3 transcripiton of PYK2 |
| F3 | Vmax13 | STAT3 transcription of c-Met |
| F4 | kc9b | c-Met phosphorylation of PYK2 |
