# Supplementary material for: Systems modelling of the EGFR-PYK2-c-Met interaction network predicts and prioritizes synergistic drug combinations for triple-negative breast cancer
Source: PLoS Comput Biol. 2018 Jun 19;14(6):e1006192. doi: 10.1371/journal.pcbi.1006192 (PMC6007894; doi:10.1371/journal.pcbi.1006192)
Supplement: S5 Table — As described in the Methods section, main text, the following qualitative constraints were incorporated into the overall objective function M for parameter estimation. (DOCX) [file pcbi.1006192.s028.docx]

**Table S5. Algebraic constraints used for parameter estimation.** As described in the Methods section, main text, the following qualitative constraints were incorporated into the overall objective function *M* for parameter estimation.

| **No** | **Biological assumption** | **Algebraic equation** | |
| --- | --- | --- | --- |
| 1 | Peak value of pEGFR by EGF stimulation > 1% of total EGFR. | $R_{1}(p)=\frac{0.01}{0.01+EGFR_{r1}(p)}$ | *EGFR_r1_*(***p***)= max(*pEGFR*(*t*,***p***))/*EGFRtotal* |
| 2 | Peak value of pPYK2 by EGF >1% of total PYK2. | $R_{2}(p)=\frac{0.01}{0.01+PYK2_{r1}(p)}$ | *PYK2_r1_*(**p**)=max(*pPYK2*(*t*,***p***))/max(*PYK2*(*t*,***p***)) |
| 3 | Peak value of pSTAT3 by EGF stimulation is >1% of total STAT3 | $R_{3}(p)=\frac{0.01}{0.01+STAT3_{r1}(p)}$ | *STAT3_r1_*(***p***)= max(*pSTAT3*(*t*,***p***))/*STAT3total* |
