# Supplementary material for: Systems modelling of the EGFR-PYK2-c-Met interaction network predicts and prioritizes synergistic drug combinations for triple-negative breast cancer
Source: PLoS Comput Biol. 2018 Jun 19;14(6):e1006192. doi: 10.1371/journal.pcbi.1006192 (PMC6007894; doi:10.1371/journal.pcbi.1006192)
Supplement: S6 Table — Out of 108 TNBC patients with available mutation information, we identified 10 patients who have reported mutations in any of the network nodes. Specifically, four patients contain EGFR mutations, two contain PYK2 mutations, another two contain STAT3 mutations, and Met and Cbl mutation were found in one single patient. The most common mutation types are SNP. However, searching for these mutations in the COSMIC mutation catalogue (http://cancer.sanger.ac.uk/cosmic), the most comprehensive mutation database currently available, we found mostly no functional effects (i.e. GOF or LOF) reported for these genetic changes, except that STAT3 H410R (indicated by an asterisk) is associated with induction of STAT3 phosphorylation and SOCS3, CCL2, JUNB and BCL3 upregulation in large granular lymphocyte (LGL) leukemia [61]. Therefore, within the EGFR-PYK2-cMET network, the majority of the considered TNBC patients displayed no functionally significant mutation events. Rather, the heterogeneity of the patient landscape primarily comes from alteration in expression levels. (DOCX) [file pcbi.1006192.s029.docx]

**Table S6. Reported mutations related to the EGFR-PYK2-cMET network within the discovery patient cohort.** Out of 108 TNBC patients with available mutation information, we identified 10 patients who have reported mutations in any of the network nodes. Specifically, four patients contain EGFR mutations, two contain PYK2 mutations, another two contain STAT3 mutations, and Met and Cbl mutation were found in one single patient. The most common mutation types are SNP. However, searching for these mutations in the COSMIC mutation catalogue (<http://cancer.sanger.ac.uk/cosmic>), the most comprehensive mutation database currently available, we found mostly no functional effects (i.e. GOF or LOF) reported for these genetic changes, except that STAT3 H410R (indicated by an asterisk) is associated with induction of STAT3 phosphorylation and SOCS3, CCL2, JUNB and BCL3 upregulation in large granular lymphocyte (LGL) leukemia [1]. Therefore, within the EGFR-PYK2-cMET network, the majority of the considered TNBC patients displayed no functionally significant mutation events. Rather, the heterogeneity of the patient landscape primarily comes from alteration in expression levels.

| **Sample id** | **Hugo**  **Symbol** | **Variant**  **Classification** | **Variant**  **Type** | **Amino Acid Change** |
| --- | --- | --- | --- | --- |
| TCGA-A2-A0T2-01 | PTK2B | Missense_Mutation | SNP | p.M957I |
| TCGA-A2-A0T0-01 | STAT3 | Silent | SNP | p.R729 |
| TCGA-BH-A18G-01 | PTK2B | Missense_Mutation | SNP | p.S606I |
| TCGA-AR-A0TU-01 | MET | In_Frame_Del | DEL | p.RS359in_frame_delP |
|  | MET | In_Frame_Del | DEL | p.A361in_frame_del |
|  | MET | Frame_Shift_Ins | INS | p.M362fs |
| TCGA-A2-A04T-01 | EGFR | Silent | SNP | p.L792 |
| TCGA-D8-A27F-01 | EGFR | Silent | SNP | p.R958 |
| TCGA-AR-A2LR-01 | EGFR | Missense_Mutation | SNP | p.E114K |
| TCGA-AO-A128-01 | STAT3 | Missense_Mutation | SNP | p.H410R* |
| TCGA-E2-A1LG-01 | CBL | Silent | SNP | p.L349 |
|  | CBL | Missense_Mutation | SNP | p.D501H |
| TCGA-D8-A1JF-01 | EGFR | Missense_Mutation | SNP | p.E114K |

**SUPPLEMENTARY REFERENCE**

1. Andersson E, Kuusanmaki H, Bortoluzzi S, Lagstrom S, Parsons A, et al. (2016) Activating somatic mutations outside the SH2-domain of STAT3 in LGL leukemia. Leukemia 30: 1204-1208.
